# Intrinsic and damage-induced JAK/STAT signaling regulate developmental timing by the *Drosophila* prothoracic gland

**DOI:** 10.1242/dmm.049160

**Published:** 2022-01-26

**Authors:** Xueya Cao, Marta Rojas, José Carlos Pastor-Pareja

**Affiliations:** 1 School of Life Sciences, Tsinghua University, Beijing 100084, China; 2 School of Medicine, Tsinghua University, Beijing 100084, China; 3 Tsinghua-Peking Center for Life Sciences, Beijing 100084, China

**Keywords:** Metamorphosis, Ring gland, Ecdysone, Tissue damage, Inflammation, SUMOylation

## Abstract

Development involves tightly paced, reproducible sequences of events, yet it must adjust to conditions external to it, such as resource availability and organismal damage. A major mediator of damage-induced immune responses in vertebrates and insects is JAK/STAT signaling. At the same time, JAK/STAT activation by the *Drosophila* Upd cytokines is pleiotropically involved in normal development of multiple organs. Whether inflammatory and developmental JAK/STAT roles intersect is unknown. Here, we show that JAK/STAT is active during development of the prothoracic gland (PG), which controls metamorphosis onset through ecdysone production. Reducing JAK/STAT signaling decreased PG size and advanced metamorphosis. Conversely, JAK/STAT hyperactivation by overexpression of pathway components or SUMOylation loss caused PG hypertrophy and metamorphosis delay. Tissue damage and tumors, known to secrete Upd cytokines, also activated JAK/STAT in the PG and delayed metamorphosis, at least in part by inducing expression of the JAK/STAT target Apontic. JAK/STAT damage signaling, therefore, regulates metamorphosis onset by co-opting its developmental role in the PG. Our findings in *Drosophila* provide insights on how systemic effects of damage and cancer can interfere with hormonally controlled development and developmental transitions.

## Abstract (precis)

**Summary:** Damage signaling from tumors mediated by JAK/STAT-activating Upd cytokines delays the *Drosophila* larva–pupa transition through co-option of a JAK/STAT developmental role in the prothoracic gland.

## INTRODUCTION

**Fig. 1.**
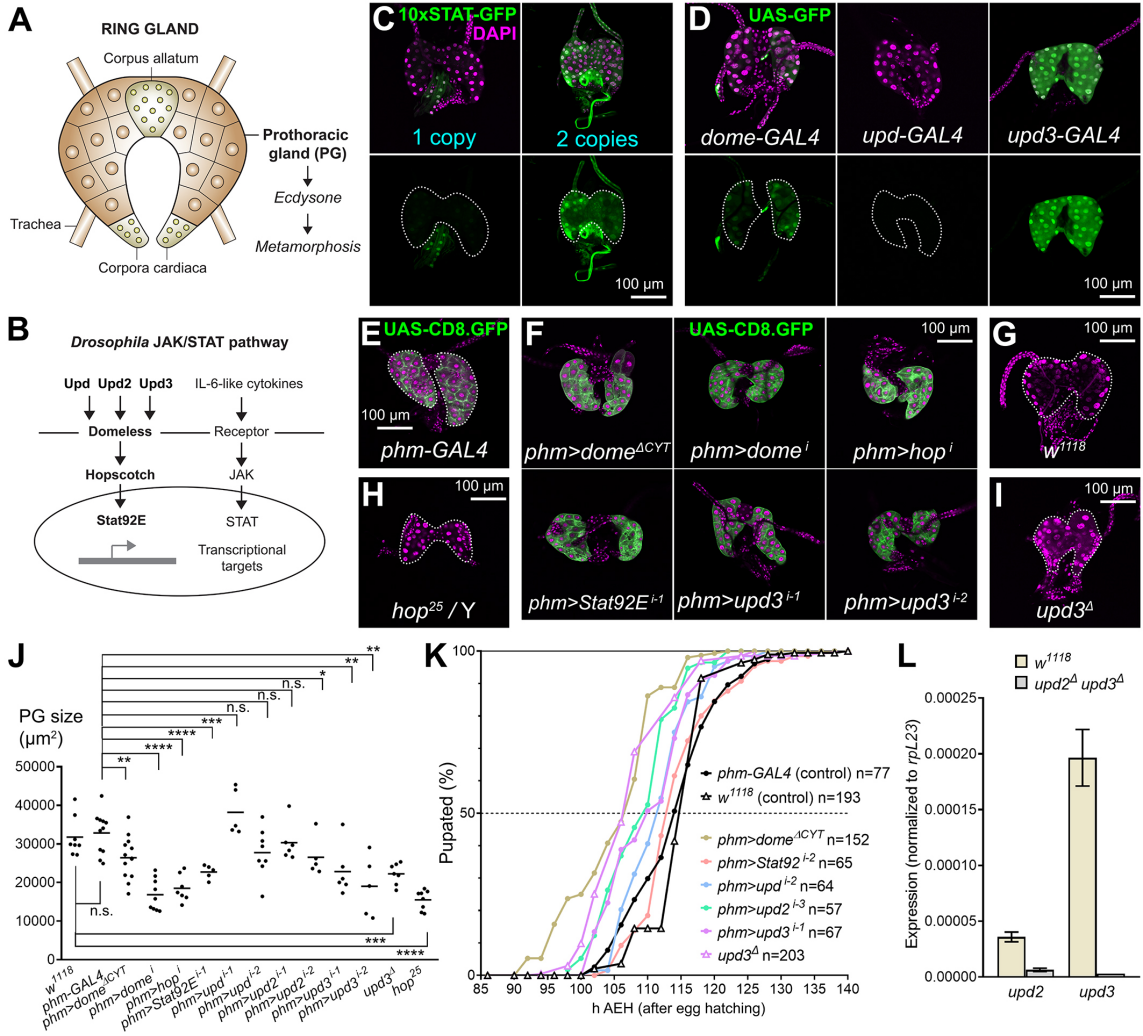
**Intrinsic JAK/STAT signaling modulates PG growth and metamorphosis.** (A) Schematic of the ring gland of a third-instar *Drosophila* larva. The prothoracic gland (PG), producing ecdysone, is the largest part of this composite neuroendocrine organ. (B) Components of the JAK/STAT signaling pathway in *Drosophila*. (C) Confocal images of ring glands dissected from wandering third-instar (wL3) larvae containing one (left) or two (right) copies of the JAK/STAT activity reporter 10xSTAT-GFP (green, separate channel in lower row). Dotted lines represent PG outline. Nuclei stained with DAPI (magenta). (D) Ring glands from wL3 larvae expressing GFP (green, separate channel in lower row) under control of GAL4 transcriptional reporters for genes encoding receptor Domeless (Dome; left), and cytokines Upd (center) and Upd3 (right). Dotted lines represent PG outline. Nuclei stained with DAPI (magenta). (E) Ring gland from a control wL3 larva expressing membrane GFP (CD8.GFP, green) under control of PG-specific *phm-GAL4*. Dotted lines represent PG outline. Nuclei stained with DAPI (magenta). (F) Ring glands from wL3 larvae expressing dominant-negative Dome^ΔCYT^ or with knockdown of *dome*, *hopscotch* (*hop*), *Stat92E* and *upd3* in the PG under control of *phm-GAL4*. Knockdown of *upd3* in *phm>upd3^i-1^* and *phm>upd3^i-2^* experiments employs different RNAi transgenes (see Table S1 for detailed genotypes in these and all other experiments throughout the paper). *phm-GAL4*-driven CD8.GFP in green. Nuclei stained with DAPI (magenta). (G) Ring gland from a *w^1118^* control wL3 larva. Dotted lines represent PG outline. Nuclei stained with DAPI (magenta). (H) Ring gland from a hypomorphic *hop^25^* mutant wL3 larva. Dotted lines represent PG outline. Nuclei stained with DAPI (magenta). (I) Ring gland from a null *upd3^Δ^* mutant wL3 larva. Dotted lines represent PG outline. Nuclei stained with DAPI (magenta). (J) Quantification of wL3 PG size in *phm-GAL4* and *w^1118^* control larvae, larvae expressing dominant-negative Dome^ΔCYT^ or with knockdown of *dome*, *hop*, *Stat92E*, *upd*, *upd2* and *upd3* under control of *phm-GAL4*, *hop^25^* mutant and *upd3^Δ^* mutant larvae. Each dot plots PG size measured as the area occupied by the PG in images of ring glands like those in E-I. Horizontal lines represent mean values. Conducted tests were Mann–Whitney tests, except for comparison of both controls and for *hop^25^*, *phm>dome^ΔCYT^*, *phm>dome^i^* and *phm>upd^i-1^* (unpaired two-tailed Student's *t*-tests). ****P<0.0001, ***P<0.001, **P<0.01, *P<0.05 and *P*>0.05 (n.s., not significant). (K) Pupation time in *phm-GAL4* and *w^1118^* controls, and in the indicated JAK/STAT loss-of-function conditions. Dot-connecting lines plot the accumulated percentage of pupated larvae over time. Time of pupation was computed as hours after egg hatching (see Materials and Methods). Number of animals examined is reported in the graph. (L) Expression of *upd2* and *upd3* in wL3 ring gland by qRT-PCR. As a control with no expression, a *upd2^Δ^ upd3^Δ^* double deletion mutant was used. Expression levels were normalized to *RpL23*. Error bars represent s.d. from three technical replicates.

The development of organisms involves internally paced, reproducible sequences of molecular and cellular events. In the development of holometabolous insects, a larval stage with little resemblance to the adult transitions to a non-feeding pupal stage during which metamorphosis takes place. This life cycle strategy arose 350 million years ago in the Carboniferous period and led to the amazing radiation of the four most successful orders of insects: coleopterans, lepidopterans, hymenopterans and dipterans (Truman and Riddiford, 2019). The temporal regulation of the larva–pupa transition depends on hormones produced by the neuroendocrine system, chief among them the steroid ecdysone, secreted by the cells of the prothoracic gland (PG) (Tennessen and Thummel, 2011; Yamanaka et al., 2013). The PG is part of the ring gland, a tripartite organ situated anterior to the brain and additionally consisting of the corpus allatum and the corpora cardiaca (Fig. 1A). The cells of the PG and corpus allatum have an ectodermal origin in tracheal primordia of the embryonic head, whereas the corpora cardiaca derive from mesoderm (Sanchez-Higueras et al., 2014). Studies in the fruit fly *Drosophila melanogaster*, a dipteran, have shown that multiple molecular signals influence ecdysone production by the PG, including prothoracicotropic hormone (Ptth), insulin, Tor, Hippo, TGFβ, EGF, Dilp8 (also known as Ilp8), nitric oxide, the circadian clock, ecdysone itself, tyramine, serotonin and Hedgehog (Hh) (Texada et al., 2020). Their coordinated actions on PG cells integrate developmental inputs and environmental cues to determine the timing of ecdysone synthesis and release. Although usually less pronounced, similar hormonally controlled developmental transitions are common in animal development. Indeed, puberty transition and insect metamorphosis might share a common Urbilaterian ancestry (Barredo et al., 2021). In general, hormonal control of developmental timing and transitions maximizes organismal fitness by ensuring development that is reproducible, robust and adjusted to conditions external to it, such as resource availability, environmental insults and damage to the organism.

Tissue damage signaling activated by mechanical wounding (Bryant, 1971; Díaz-García and Baonza, 2013) or high levels of cell death (Akai et al., 2021; Hackney et al., 2012; Simpson and Schneiderman, 1975; Smith-Bolton et al., 2009; Stieper et al., 2008) is well known to delay the onset of metamorphosis. Damage-induced extension of the larval period may have evolved to allow for more complete healing and regeneration before transition to the next developmental stage occurs (Hariharan and Serras, 2017). Tumorous growth perturbations, sharing tissue damage inflammatory mechanisms with wounds (Pastor-Pareja et al., 2008; Pastor-Pareja and Xu, 2013), delay or completely inhibit the larva–pupa transition as well (Menut et al., 2007; Pagliarini and Xu, 2003; Sehnal and Bryant, 1993). One of the signals mediating damage-induced metamorphosis delay is the biosynthesis of retinoids (Halme et al., 2010). Through poorly understood mechanisms, retinoids inhibit production in the central nervous system of Ptth, required for ecdysone production by the PG. Another signal acting on PG ecdysone production through inhibition of Ptth synthesis is Dilp8, a member of the insulin-like/relaxin family of peptide hormones. Dilp8 is produced by damaged tissues and tumors downstream of the c-Jun N-terminal kinase (JNK; also known as Bsk) pathway (Colombani et al., 2012; Garelli et al., 2012). Autocrine Janus kinase/Signal transducer and activator of transcription (JAK/STAT) signaling, activated in wounds and tumors by JNK-induced expression of Unpaired (Upd) cytokines (Pastor-Pareja et al., 2008; Wu et al., 2010), has been reported to enhance local Dilp8 production by the damaged tissue (Katsuyama et al., 2015). In the tissue damage response, however, JAK/STAT-activating Upd cytokines have been shown to act not just locally, driving damage-induced regenerative growth (Katsuyama et al., 2015; La Fortezza et al., 2016; Santabarbara-Ruiz et al., 2015; Wu et al., 2010), but also systemically. Indeed, damaged-induced Upd cytokines mediate an innate immune response that induces proliferation of circulating macrophages and amplifies itself through additional Upd cytokine expression in the fat body (Pastor-Pareja et al., 2008). Furthermore, Upd cytokines have been shown to act systemically as well in inter-organ communication of nutritional status in the adult (Rajan and Perrimon, 2012). Additional, non-local roles of damage-induced Upd cytokines in developmental timing are therefore possible.

The JAK/STAT signaling pathway (Fig. 1B) is a major mediator of innate immune responses in vertebrates and insects alike (Agaisse and Perrimon, 2004; Lemaitre and Hoffmann, 2007). In *Drosophila*, JAK/STAT signaling is activated by three highly similar Interleukin-6-related cytokine ligands: Upd (also known as Upd1) (Harrison et al., 1998), Upd2 (Gilbert et al., 2005; Hombría et al., 2005) and Upd3 (Agaisse et al., 2003). Upd cytokines bind to the transmembrane receptor Domeless (Dome) (Brown et al., 2001; Chen et al., 2002), inducing activation of the receptor-associated JAK homolog Hopscotch (Hop) (Binari and Perrimon, 1994). Activated Hop in turn phosphorylates the STAT transcription factor homolog Stat92E (Hou et al., 1996; Yan et al., 1996), which then translocates as a dimer to the nucleus to activate expression of target genes (Arbouzova and Zeidler, 2006; Rawlings et al., 2004; Zeidler and Bausek, 2013). In addition to its immune roles, JAK/STAT activation by Upd cytokines is pleiotropically involved in normal development and differentiation of multiple non-immune organs and cell types in *Drosophila*. Examples of non-immune contexts in which JAK/STAT signaling is known to play a developmental role include segmental patterning (Binari and Perrimon, 1994), tracheae (Brown et al., 2001), border cells of the ovarian follicle (Beccari et al., 2002; Silver and Montell, 2001), the eye (Bach et al., 2003; Zeidler et al., 1999), the wing (Rodrigues et al., 2012), the optic lobe (Yasugi et al., 2008) and multiple stem cell niches (Herrera and Bach, 2019). A systemic JAK/STAT immune response, such as the tissue damage response, may therefore influence the development of multiple tissues. Whether the inflammatory and developmental functions of JAK/STAT signaling intersect, however, has not been investigated.

Here, we studied the role of JAK/STAT signaling in the PG. We found that JAK/STAT signaling is active during normal PG development. Reduced JAK/STAT signaling decreased average PG size and slightly advanced metamorphosis, while pathway hyperactivation caused PG hypertrophy and delayed the larva–pupa transition. Tissue damage and tumors, known to secrete Upd cytokines, also led to JAK/STAT activation in the PG and metamorphosis delay. JAK/STAT effects on the PG, our results indicate, are mediated at least in part by downstream expression of Apontic (Apt). Altogether, our experiments reveal that damage signaling by Upd cytokines regulates the onset metamorphosis by directly activating JAK/STAT signaling in the PG, thus co-opting a developmental role of JAK/STAT therein.

## RESULTS

### Intrinsic JAK/STAT signaling modulates PG growth and metamorphosis

To investigate a possible role of JAK/STAT signaling in ring gland development and developmental timing, we examined expression of 10xSTAT-GFP, a reporter of JAK/STAT signaling in which GFP expression is driven by ten copies of the STAT binding sequence from an intron of JAK/STAT target *Socs36E* (Bach et al., 2007). In ring glands dissected from wandering third-instar (wL3) larvae, we were able to detect expression of the STAT-GFP reporter in the PG, increased when two copies of the reporter were present in homozygous flies (Fig. 1C; see also Fig. S1A,B for a time course of STAT-GFP and STAT-dGFP reporters during larval development). In addition, we found that reporters *dome-GAL4* and *upd3-GAL4*, transcriptional reporters for the expression of *dome*, encoding the JAK/STAT receptor, and *upd3*, encoding one of the three JAK/STAT-activating, Interleukin-6-related cytokines present in *Drosophila*, were expressed in the PG as well (Fig. 1D; see also Fig. S1C,D for a time course of these reporters during larval development), further suggesting that JAK/STAT signaling is active in the PG. To test a role of JAK/STAT signaling in the PG, we examined ring glands in different conditions affecting JAK/STAT signaling. Knockdown of the genes encoding receptor Dome, the kinase Hop and transcription factor Stat92E under control of PG-specific *phm-GAL4*, as well as expression of dominant-negative Dome (Dome^ΔCYT^), resulted in ring glands that were, on average, smaller than controls (Fig. 1E,F, quantified in J). Among the three JAK/STAT ligands, knockdown of *upd3* with two different RNAi transgenes showed a consistent decrease in ring gland size (Fig. 1E,F,J), while knockdown of *upd* and *upd2* had non-significant or less significant effects. Furthermore, null *upd3* (Osman et al., 2012) and hypomorphic *hop* mutants (Perrimon and Mahowald, 1986) also showed significantly reduced PG size (Fig. 1G-I). Because the PG has a major role in regulating developmental timing, we next investigated the effect of JAK/STAT signaling reduction on the timing of the larva–pupa transition. To do that, we recorded the time of pupation of animals after egg hatching [embryo–first-instar (L1) transition]. Compared to controls, we observed that the time of the larva–pupa transition was advanced in different JAK/STAT loss-of-function conditions, with *upd3* null mutants and Dome^ΔCYT^ expression in the PG showing the clearest such advanced pupation effects (Fig. 1K). Through quantitative real-time PCR, and using a *upd2 upd3* double deletion mutant as a control, we confirmed that *upd3* is expressed in the ring gland and *upd2* to a lesser extent (Fig. 1L). In all, these results indicate that intrinsically activated JAK/STAT signaling functions in the PG, where it contributes to its development and modulates the timing of the larva–pupa transition.

### JAK/STAT hyperactivation in the PG delays metamorphosis

**Fig. 2.**
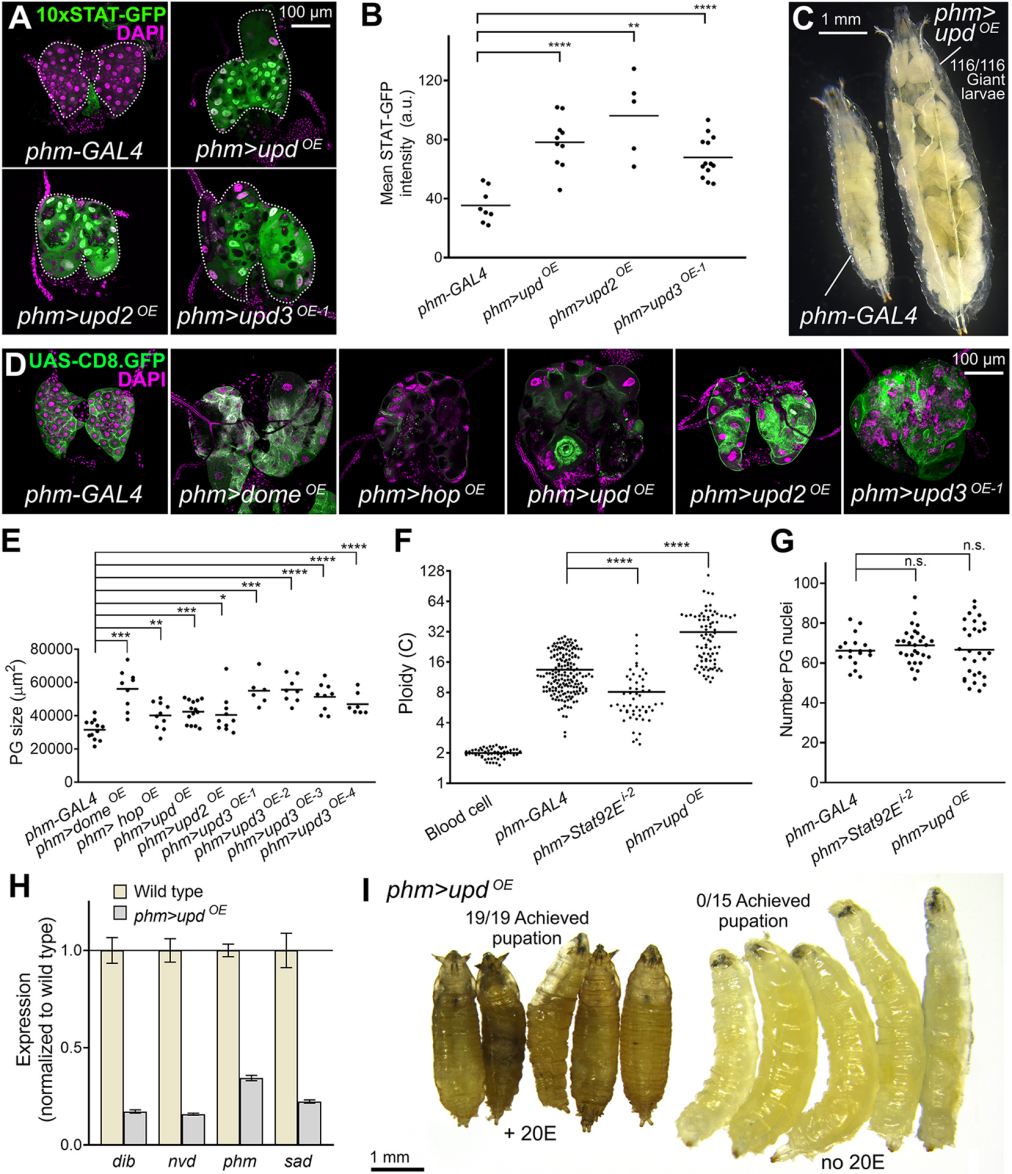
**JAK/STAT hyperactivation causes PG hypertrophy and delayed metamorphosis.** (A) Expression of JAK/STAT activity reporter 10xSTAT-GFP (green, separate channel in lower row) in the PG of control *phm-GAL4* wL3 larvae and giant larvae overexpressing *upd*, *upd2* and *upd3* under control of *phm-GAL4*. Dotted lines represent PG outline. Nuclei stained with DAPI (magenta). (B) Quantification of 10xSTAT-GFP intensity in PG of the larvae overexpressing *upd*, *upd2* and *upd3* under *phm-GAL4* control. Each dot plots mean GFP intensity (total fluorescence intensity divided by area) in the PG measured in images like those in A. Horizontal lines represent mean values. Significance of differences in unpaired two-tailed Student's *t*-tests (*phm>upd^OE^* and *phm>upd3^OE^*) and Mann–Whitney tests (*phm>upd2^OE^*) is reported. a.u., arbitrary units. (C) Control *phm-GAL4* wL3 larva (left) and larva overexpressing *upd* under *phm-GAL4* control in the PG (*phm>upd^OE^*, right). Unable to pupate, *phm>upd^OE^* third-instar (L3) larvae indefinitely extend their larval period and become giant larvae. (D) Ring glands from a control *phm-GAL4* wL3 larva and from L3 giant larvae overexpressing *dome*, *hop*, *upd*, *upd2* and *upd3* in the PG under control of *phm-GAL4*. *phm-GAL4*-driven CD8.GFP in green. Nuclei stained with DAPI (magenta). (E) Quantification of PG size in wL3 *phm-GAL4* control larvae and in giant larvae overexpressing JAK/STAT pathway components in the PG under control of *phm-GAL4* as indicated. Each dot plots PG size measured from images like those in D. Horizontal lines represent mean values. Significance of differences in statistical tests is reported. Conducted tests were unpaired two-tailed Student's *t*-tests (*phm>hop^OE^*, *phm>upd^OE^*, *phm>upd3^OE-2^* and *phm>upd3^OE-3^*), unpaired two-tailed *t*-tests with Welch's correction (*phm>dome^OE^*) and Mann–Whitney tests (*phm>upd2^OE^*, *phm>upd3^OE-1^* and *phm>upd3^OE-4^*). (F) Quantification of ploidy in PG nuclei of control *phm-GAL4* wL3 larvae, *phm>Stat92E^i-2^* wL3 larvae and giant *phm>upd^OE-1^* larvae. Each dot plots ploidy of a nucleus calculated based on DNA content (DAPI intensity) integrated from confocal stacks of the whole PG and with reference to DNA content in diploid blood cells (see Materials and Methods). *n*=58, 172, 53 and 80 nuclei for diploid blood cells, control *phm-GAL4* wL3 larvae, *phm>Stat92E*^*i-2*^ wL3 larvae and giant *phm>upd*^*OE-1*^ larvae respectively. Horizontal lines represent mean values. Significance of differences in Mann–Whitney tests is reported. (G) Number of nuclei in *phm-GAL4* wL3 larvae, *phm>Stat92E^i-2^* wL3 larvae and giant *phm>upd^OE^* larvae. Each point represents the number of PG nuclei in one ring gland as counted manually from confocal stacks of the whole PG. *n*=18, 29 and 29, respectively. Differences were not significant in unpaired two-tailed Student's *t*-tests (*phm>Stat92E^i-2^*) and Mann–Whitney tests (*phm>upd^OE^*). (H) Quantification of expression of Halloween genes of the ecdysone synthesis pathway by qRT-PCR. Expression levels relative to *RpL23* were normalized to the wild-type *phm-GAL4* control. Representative results are shown of three biological replicates, all demonstrating strong downregulation. Error bars represent s.d. of three technical replicates. (I) Rescue of pupation in *phm>upd^OE^* larvae after 20-hydroxyecdysone (20E) treatment (see Materials and Methods). ****P<0.0001, ***P<0.001, **P<0.01, *P<0.05 and *P*>0.05 (n.s., not significant).

To further investigate the role of JAK/STAT signaling in the PG, we aimed at increasing JAK/STAT activity and studying its effects on PG development and metamorphosis onset. To do that, we overexpressed JAK/STAT cytokines Upd, Upd2 and Upd3 in the PG under control of *phm-GAL4*. We found that expression of all three cytokines showed similar ability to activate JAK/STAT signaling, as evidenced by highly increased expression of the 10xSTAT-GFP activity reporter (Fig. 2A, quantified in B). In addition to this, we observed that larvae expressing JAK/STAT cytokines in their PG did not pupate and became giant larvae (Fig. 2C). The PG in these larvae, and in larvae overexpressing Dome and Hop in their PG, became highly enlarged and abnormal, with some cells presenting a large degree of vacuolation (Fig. 2D,E; see also Fig. S2), suggestive of a degenerative or autolytic process. PG hypertrophy was accompanied by an increase in ploidy of these cells estimated by their DNA content, while average PG ploidy was reduced upon knockdown of *Stat92E* (Fig. 2F). The number of PG nuclei in JAK/STAT loss (*phm>Stat92E^i^*) and gain (*phm>upd^OE^*) conditions, however, did not change with respect to the wild type (Fig. 2G). Consistent with their inability to undergo larva–pupa transition, quantitative real-time PCR showed that the PG of animals overexpressing Upd expressed reduced levels of *disembodied* (*dib*), *neverland* (*nvd*), *phantom* (*phm*) and *shadow* (*sad*), encoding Halloween group enzymes, involved in the synthesis of ecdysone (Fig. 2H). Furthermore, pupation of *phm>upd^OE^* animals could be rescued by supplementing 20-hydroxyecdysone (20E) (Fig. 2I). All these results support a positive role of JAK/STAT signaling in PG cell growth and endoreplication. At the same time, they indicate that whereas basal levels of JAK/STAT signaling counter premature metamorphosis, JAK/STAT hyperactivation has the opposite effect and prevents pupation.

**Fig. 3.**
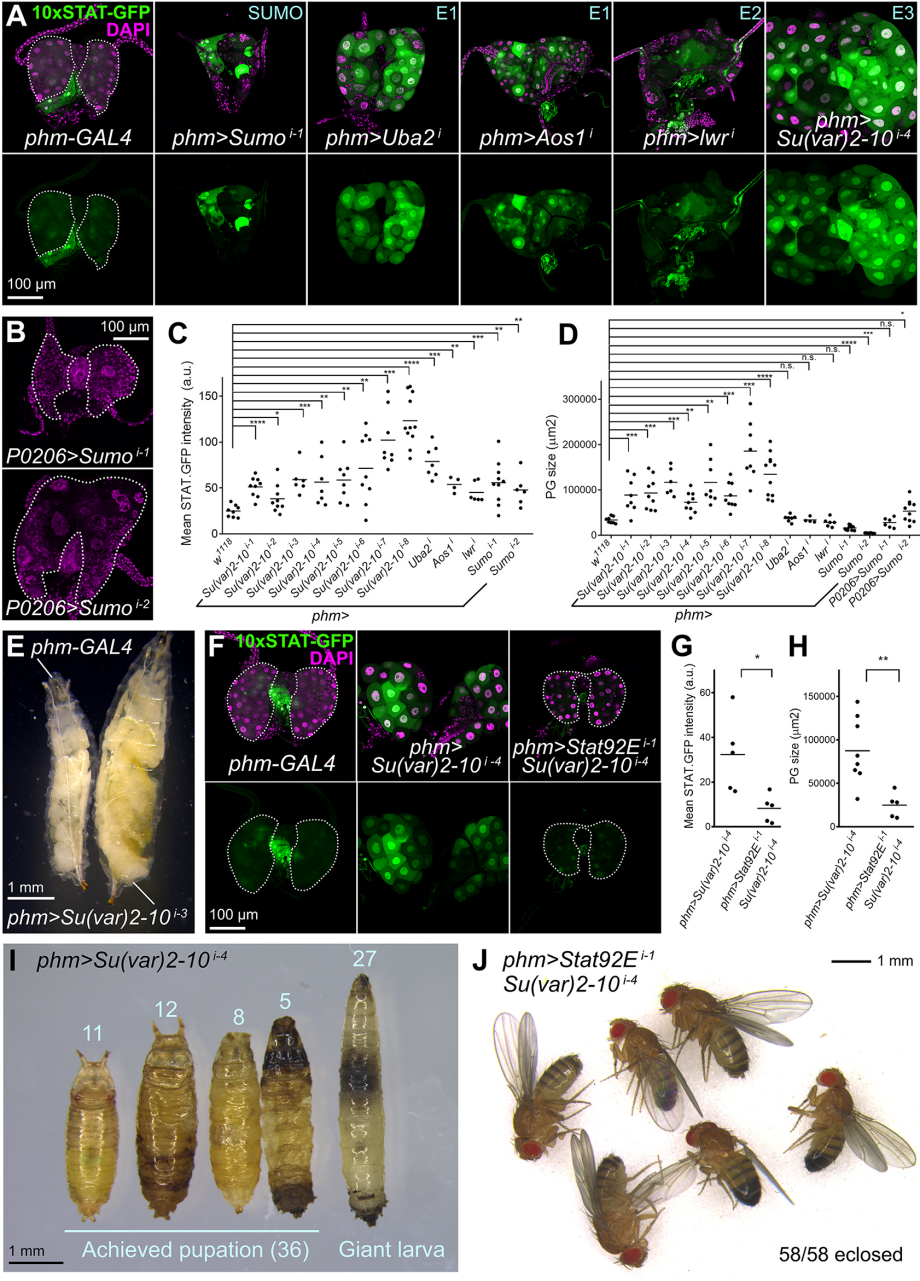
**SUMOylation prevents JAK/STAT hyperactivation during normal PG development.** (A) Expression of JAK/STAT activity reporter 10xSTAT-GFP (green, separate channel in lower row) in the PG of control *phm-GAL4* wL3 larvae and giant larvae in which expression of SUMOylation pathway components has been knocked down under control of *phm-GAL4*. Dotted lines represent PG outline. Nuclei stained with DAPI (magenta). (B) Ring glands from wL3 larvae in which *Sumo* (previously called *smt3*) is knocked down under control of *P0206-GAL4*. *P0206>Sumo^i-1^* and *P0206>Sumo^i-2^* experiments employ different RNAi transgenes (see Table S1). Dotted lines represent PG outline. Nuclei stained with DAPI (magenta). (C) Quantification of 10xSTAT-GFP intensity in PG of control *phm-GAL4* wL3 larvae and giant larvae in which expression of SUMOylation pathway components has been knocked down under control of *phm-GAL4*. Each dot plots mean GFP intensity in the PG measured in images like those in A. Horizontal lines represent mean values. Significance of differences in statistical tests is reported. Conducted tests were unpaired two-tailed *t*-tests with Welch's correction except for *phm>Su(var)2-10^i-1^* (unpaired two-tailed Student's *t*-test), *phm>Su(var)2-10^i-3^*, *phm>Su(var)2-10^i-4^*, *phm>Uba2^i^*, *phm>Aos1^i^*, *phm>lwr^i^*, and *phm>Sumo^i-2^* (Mann–Whitney tests). (D) Quantification of PG size in control *phm-GAL4* wL3 larvae and giant larvae in which expression of SUMOylation pathway components has been knocked down under control of *phm-GAL4*. Each dot plots PG size measured from images like those in A and B. Horizontal lines represent mean values. Significance of differences in statistical tests is reported. Conducted tests were unpaired two-tailed *t*-tests with Welch's correction except for *phm>Sumo^i-1^* (unpaired two-tailed Student's *t*-test), and *phm>Su(var)2-10^i-3^*, *phm>Uba2^i^*, *phm>Aos1^i^*, *phm>lwr^i^*, *phm>Sumo^i-2^*, and *P0206>Sumo^i-1^* (Mann–Whitney tests). (E) Control *phm-GAL4* wL3 larva (left) and giant *phm>Su(var)2-10^i-3^* larva (right). (F) Expression of JAK/STAT activity reporter 10xSTAT-GFP (green, separate channel in lower row) in the PG of control *phm-GAL4* wL3 larvae (left) and larvae in which expression of *Su(var)2-10* (center) and both *Su(var)2-10* and *Stat92E* (right) has been knocked down under control of *phm-GAL4*. Dotted lines represent PG outline. Nuclei stained with DAPI (magenta). (G) Quantification of 10xSTAT-GFP intensity in PG of wL3 larvae in which expression of *Su(var)2-10* and both *Su(var)2-10* and *Stat92E* have been knocked down under control of *phm-GAL4*. Each dot plots mean GFP intensity in the PG measured from images like those in F. Horizontal lines represent mean values. Difference was significant in a Mann–Whitney test. (H) Quantification of PG size in wL3 larvae in which expression of *Su(var)2-10* and both *Su(var)2-10* and *Stat92E* have been knocked down under control of *phm-GAL4*. Each dot plots PG size measured in images like those in F. Horizontal lines represent mean values. Difference was significant in a Mann–Whitney test. (I) Defective pupation in *phm>Su(var)2-10^i-4^* larvae. Number of animals unable to pupate (giant larvae) and exhibiting pupation defects is indicated. Even if pupation was achieved, adults did not eclose. (J) *phm>Su(var)2-10^i-4^* animals in which *Stat92E* is additionally knocked down reach adulthood. ****P<0.0001, ***P<0.001, **P<0.01, *P<0.05 and *P*>0.05 (n.s., not significant).

We were intrigued by the JAK/STAT hyperactivation PG phenotype, combining enlargement and partial tissue destruction. We found in the literature a strikingly similar phenotype caused by loss of function of *Sumo* (previously named *smt3*), encoding the *Drosophila* homolog of Ubiquitin-like post-translational modifier SUMO (Talamillo et al., 2008). Furthermore, the E3 SUMO ligase Su(var)2-10, also known as PIAS, is known to negatively regulate STAT activity in *Drosophila* and humans (Betz et al., 2001; Grönholm et al., 2010). We found that knockdown of *Sumo*, *Su(var)2-10* and other components of the *Drosophila* SUMOylation cascade strongly increased JAK/STAT activity in the ring gland, evidenced by increased 10xSTAT-GFP reporter expression (Fig. 3A, quantified in C). Extreme PG hypertrophy was observed with RNA interference (RNAi) transgenes targeting *Su(var)2-10* (Fig. 3A, quantified in D), producing in addition inhibition of the larva–pupa transition (Fig. 3E). In contrast to *Su(var)2-10*, knockdown of *Sumo* resulted in PGs that were smaller than those of the wild type (Fig. 3A,D). This suggests that loss of *Sumo* may impair regulation of all SUMOylated proteins, including some for which upregulation affects cell growth or viability, whereas more upstream components up to the E3 conjugating enzyme Su(var)2-10 will affect increasingly specific SUMOylation target subsets. Nonetheless, we could confirm with the alternative ring gland driver *P0206-GAL4* that knockdown of Sumo can itself enlarge the PG (Fig. 3B,D), as reported originally by Talamillo et al. (2008). Showing that suppression of SUMOylation affects PG development through JAK/STAT hyperactivation, knockdown of *Stat92E* strongly suppressed JAK/STAT activity and hypertrophy in the PG induced by *Su(var)2-10* knockdown (Fig. 3E, quantified in F,G). Furthermore, *Stat92E* knockdown allowed development of these animals into adults (Fig. 3H,I). These results indicate that JAK/STAT activity in the PG is negatively regulated by the SUMOylation cascade and confirm the potential of the JAK/STAT pathway to influence PG development and timing of metamorphosis.

### Tumors and tissue damage activate PG-extrinsic JAK/STAT signaling to delay metamorphosis

**Fig. 4.**
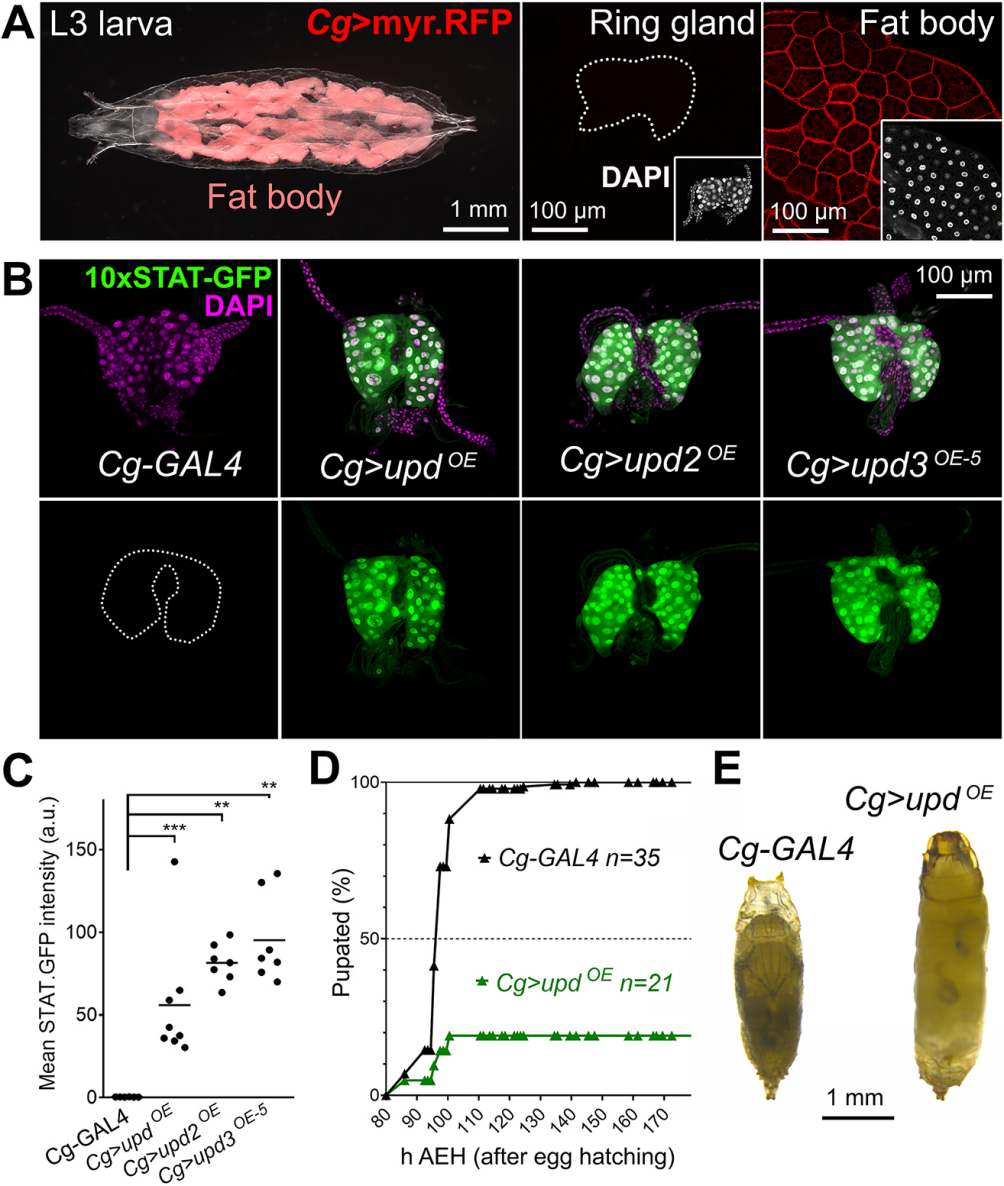
**Extrinsically produced Upd cytokines can activate JAK/STAT signaling in the PG.** (A) wL3 larva (left) showing myr.RFP expression driven by *Cg-GAL4* (red) in the fat body (right), but not in the ring gland (center). White light and red fluorescence images have been superimposed in the left panel. Insets show ring gland and fat body nuclei stained with DAPI (white). Dotted lines represent ring gland outline. (B) Expression of JAK/STAT activity reporter 10xSTAT-GFP (green, separate channel in lower row) in the PG of control *Cg-GAL4* wL3 larvae and giant larvae overexpressing *upd*, *upd2* and *upd3* in the fat body under control of Cg-GAL4. Dotted lines represent PG outline. Nuclei stained with DAPI (magenta). (C) Quantification of 10xSTAT-GFP intensity in PG of control *Cg-GAL4* wL3 larvae and giant larvae overexpressing *upd*, *upd2* and *upd3* in the fat body under control of Cg-GAL4. Each dot plots mean GFP intensity in the PG measured in images like those in B. Horizontal lines represent mean values. Significance of differences in Mann–Whitney tests is reported. ***P<0.001 and **P<0.01. (D) Pupation time in *Cg-GAL4* control and *Cg>upd^OE^* animals. Dot-connecting lines plot the accumulated percentage of pupated larvae over time. (E) Control *Cg-GAL4* pupa (left) and pupa of an animal overexpressing *upd* under control of *Cg-GAL4* in the fat body (right). Even if pupation is achieved, no adults eclose from *Cg>upd^OE^* pupae.

Upd cytokines are abundantly produced in innate immune responses (Agaisse and Perrimon, 2004). Of note, Upd cytokines produced by tumors or wounds have been previously shown to activate JAK/STAT signaling in the fat body and blood cells, causing a systemic response through fat body amplification of cytokine production (Pastor-Pareja et al., 2008). Because we had found that JAK/STAT activity regulated pupation time in the PG, we hypothesized that extrinsically produced Upd cytokines could act in the PG similarly to intrinsically expressed ones. To test this, we overexpressed cytokines Upd, Upd2 and Upd3 outside the PG under control of *Cg-GAL4*, expressed in the fat body and circulating blood cells of the hemolymph-filled body cavity, but not in the ring gland (Fig. 4A). In all three cases, we found that PG-extrinsic expression of Upd cytokines was capable of inducing high levels of 10xSTAT-GFP reporter expression in the PG (Fig. 4B, quantified in C). Furthermore, these animals showed absent or delayed metamorphosis (Fig. 4D,E). These results, importantly, show that Upd cytokines produced outside the PG can activate JAK/STAT signaling and produce developmental delay, similar to that observed when Upd cytokines were overexpressed by PG cells or when activity of the pathway was manipulated inside the PG.

**Fig. 5.**
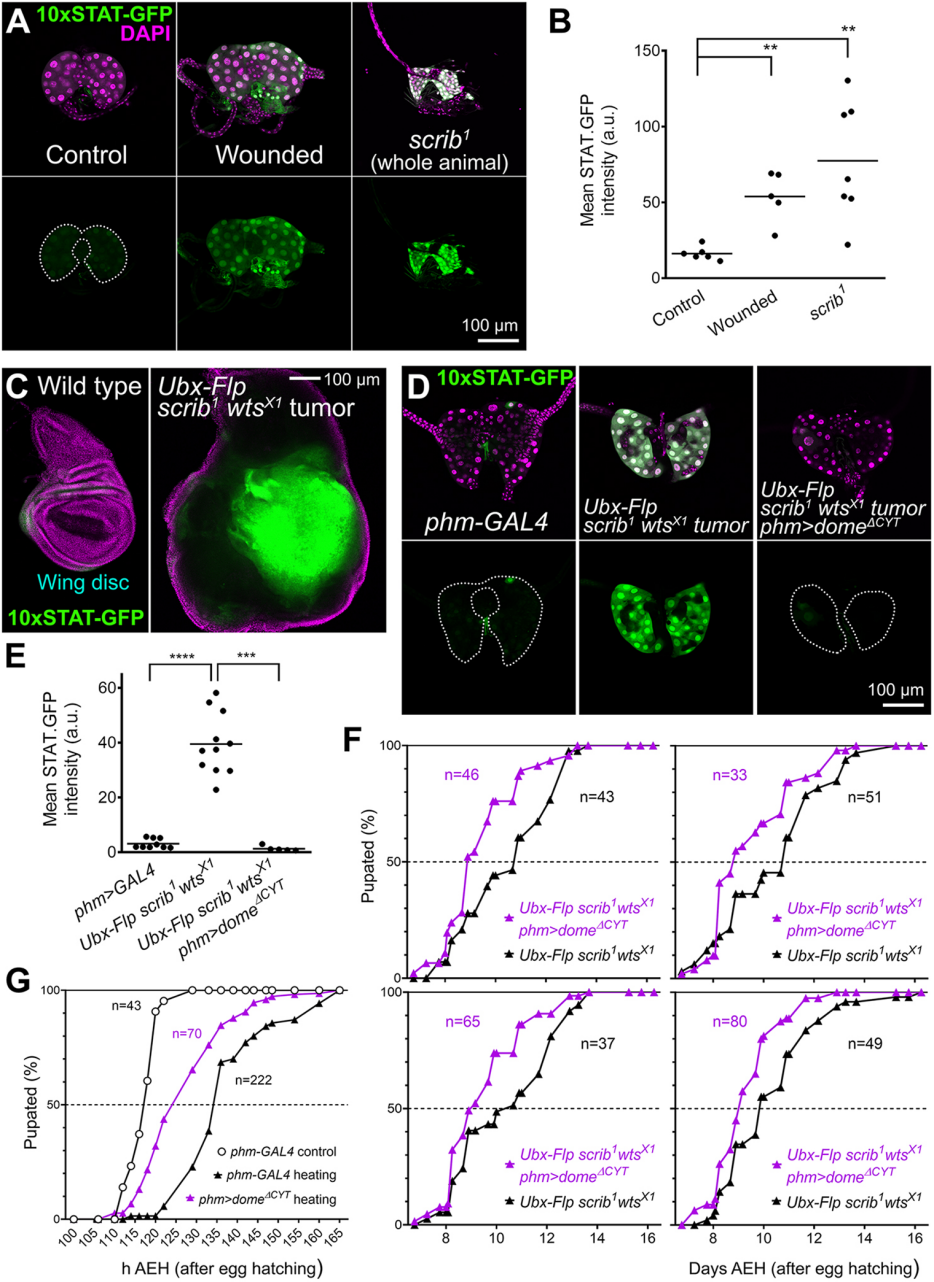
**Tumors and tissue damage activate PG-extrinsic JAK/STAT signaling to delay metamorphosis.** (A) Expression of JAK/STAT activity reporter 10xSTAT-GFP (two copies, green, separate channel in lower row) in the PG of control (left) and puncture-wounded (center) wL3 larvae, and of tumor-containing *scrib^1^* giant larvae (right). Dotted lines represent PG outline. Nuclei stained with DAPI (magenta). (B) Quantification of 10xSTAT-GFP intensity in PG of control and puncture-wounded wL3 larvae, and of tumor-containing *scrib^1^* giant larvae. Each dot plots mean GFP intensity in the PG measured in images like those in A. Horizontal lines represent mean values. Differences in Mann–Whitney tests were significant. (C) Expression of JAK/STAT activity reporter 10xSTAT-GFP (green, separate channel in lower row) in a control wL3 wing imaginal disc (left) and in a wing disc from a wL3 larva containing *scrib^1^ wts^X1^* tumor clones induced through Ubx-Flp-driven mitotic recombination (right). Nuclei stained with DAPI (magenta). (D) Expression of JAK/STAT activity reporter 10xSTAT-GFP (green, separate channel in lower row) in the wL3 PG of control *phm-GAL4* larvae (left), larvae with *scrib^1^ wts^X1^* tumor clones in the wing disc (center) and larvae with *scrib^1^ wts^X1^* wing tumors additionally expressing Dome^ΔCYT^ in the PG under *phm-GAL4* control (right). Dotted lines represent PG outline. Nuclei stained with DAPI (magenta). (E) Quantification of 10xSTAT-GFP intensity in the wL3 PG of control *phm-GAL4* larvae, larvae with *scrib^1^ wts^X1^* wing tumors and larvae with *scrib^1^ wts^X1^* tumors additionally expressing Dome^ΔCYT^ in the PG under *phm-GAL4* control. Each dot plots mean GFP intensity in the PG measured in images like those in D. Horizontal lines represent mean values. Significance of differences in statistical tests is reported. Conducted tests were unpaired two-tailed *t*-test with Welch's correction (control versus *scrib^1^ wts^X1^* tumor) and Mann–Whitney test (*scrib^1^ wts^X1^* tumor versus *scrib^1^ wts^X1^* tumor expressing Dome^ΔCYT^ in the PG). (F) Pupation time in larvae with *scrib^1^ wts^X1^* wing tumors and larvae with *scrib^1^ wts^X1^* tumors additionally expressing Dome^ΔCYT^ in the PG under *phm-GAL4* control. Four repeats of the experiment are shown. Dot-connecting lines plot the accumulated percentage of pupated larvae over time. (G) Pupation time in *phm-GAL4* control animals and in both *phm-GAL4* and *phm>dome^ΔCYT^* animals heated at 39°C for 2.5 h during the mid-L3 stage. Dot-connecting lines plot accumulated percentage of pupated larvae over time. ****P<0.0001, ***P<0.001 and **P<0.01.

Having shown that extrinsically produced Upd cytokines can activate JAK/STAT signaling in the PG, we asked whether tissue damage conditions elevating systemic Upd cytokine levels induced JAK/STAT activity in the PG. We found that puncture wounding of the larval epidermis as well as neoplastic tumors in *scribbled* (*scrib*) mutants increased JAK/STAT activity in the PG (Fig. 5A, quantified in B), suggesting that, upon tissue damage, JAK/STAT activation in the PG could contribute to metamorphosis delay in these conditions. To test this, we employed a mosaic model of clonally induced tumors in the wing imaginal discs. In this model, large tumors formed by *scribbled warts* (*scrib wts*) homozygous double mutant cells are generated through mitotic recombination in a wild-type animal (Fig. 5C). Despite pupation delay by *scrib wts* wing disc tumors, all animals in the end are capable of undergoing metamorphosis. In this tumor model, we found that activation of JAK/STAT signaling in the PG was decreased by expression of dominant-negative receptor Dome^ΔCYT^ (Fig. 5D, quantified in E). Furthermore, Dome^ΔCYT^ expression in the PG partially rescued pupation delay in tumor-bearing animals (Fig. 5F, four repeats of the same experiment). Similarly, Dome^ΔCYT^ expression in the PG partially rescued metamorphosis delay in an experiment in which damage was induced by heating mid-third-instar (L3) larvae at 39°C for 2.5 h (Fig. 5G). Altogether, these results show that JAK/STAT activation in the PG extrinsically induced by tumors and tissue damage contributes to delaying the larva–pupa developmental transition.

### JAK/STAT activation upregulates transcription factor Apt and miRNA *bantam* (*ban*) in the PG

**Fig. 6.**
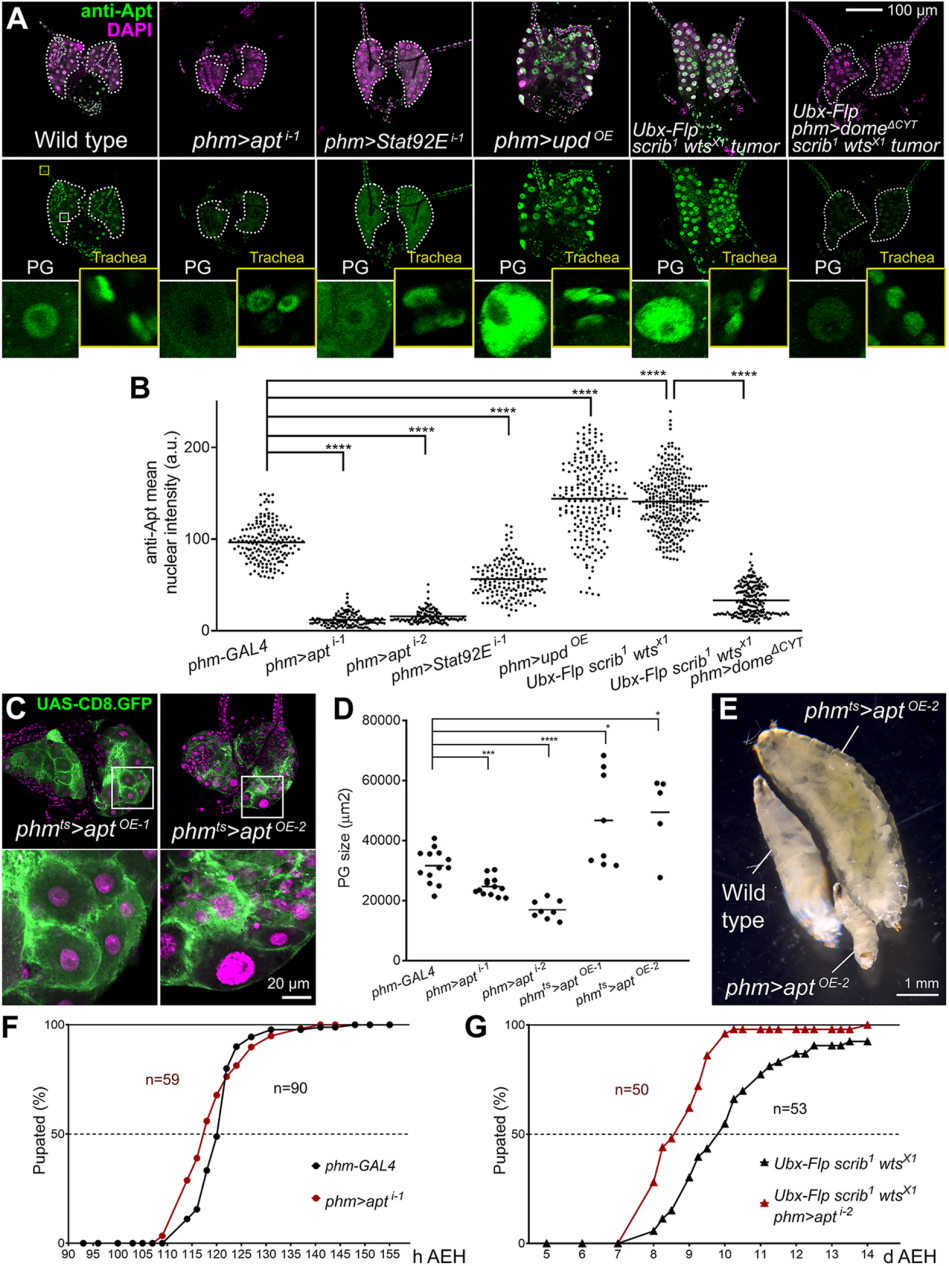
**The JAK/STAT target Apontic (Apt) induces PG hypertrophy and metamorphosis delay.** (A) Anti-Apt antibody stainings (green, separate channel in lower row) of wL3 ring glands of the indicated genotypes. Dotted lines represent PG outline. Nuclei stained with DAPI (magenta). Higher-magnification insets show Apt expression in PG nuclei and in tracheal nuclei as an internal control in which levels do not change. (B) Quantification of anti-Apt signal in wL3 PG from larvae of the indicated genotypes. Each dot plots mean GFP intensity in a PG nucleus measured in images like those in A. Horizontal lines represent mean values. *n*=178, 122, 112, 173, 226, 320 and 202 nuclei, respectively. Significance of differences in statistical tests is reported. Conducted tests were Mann–Whitney tests, except for *phm-GAL4* control versus *phm>upd^OE^* and *scrib^1^ wts^X1^* tumors (unpaired two-tailed *t*-tests with Welch's correction). (C) Ring glands from L3 giant larvae overexpressing *apt* in the PG under control of *phm-GAL4* and *tub-GAL80^ts^*. *phm-GAL4*-driven CD8.GFP in green. Animals were transferred at L2 stage from 18°C to 30°C to initiate *apt* overexpression. Overexpression of *apt* in *phm^ts^>apt^OE-1^* and *phm^ts^>apt^OE-2^* employs different transgenes (see Table S1). Higher-magnification insets (white squares) are shown in the lower row. Nuclei stained with DAPI (magenta). (D) Quantification of PG size in control *phm-GAL4* wL3 larvae, *phm>apt^i^* wL3 larvae and giant *phm^ts^>apt^OE^* larvae. Each dot plots PG size measured in images like those in A and C. Horizontal lines represent mean values. Significance of differences in statistical tests is reported. Conducted tests were unpaired two-tailed Student's *t*-test (*phm>apt^i-1^*, *phm>apt^i-2^*), unpaired two-tailed *t*-test with Welch's correction (*phm^ts^>apt^OE-1^*) and Mann–Whitney test (*phm^ts^>apt^OE-2^*). (E) Control *phm-GAL4* wL3 larva, *phm>apt^OE-2^* L2 larva and giant *phm^ts^>apt^OE-2^* L3 larva (L2 temperature shift). (F) Pupation time in *phm-GAL4* control and *phm>apt^i-1^* animals. Dot-connecting lines plot accumulated percentage of pupated larvae over time. (G) Pupation time in larvae with *scrib^1^ wts^X1^* wing tumors and larvae with *scrib^1^ wts^X1^* tumors and additional knockdown of *apt* in the PG under *phm-GAL4* control. Dot-connecting lines plot the accumulated percentage of pupated larvae over time. Number of animals examined is reported in the graph. ****P<0.0001, ***P<0.001 and *P<0.05.

To further investigate the action of JAK/STAT in the ring gland, we searched for known targets of JAK/STAT signaling that were expressed in the PG. We found one such candidate in Apt, highly present in the PG according to published images (Rodrigues et al., 2021). Apt, also known as Tracheae defective, is a transcription factor known to act as a downstream target of JAK/STAT in border cell migration (Starz-Gaiano et al., 2008). It is also involved in the development of multiple tissues, including the tracheal system (Eulenberg and Schuh, 1997). Staining with an anti-Apt antibody (Liu et al., 2014) confirmed expression of Apt in the PG and the entire ring gland (Fig. 6A). Furthermore, expression of Apt was highly upregulated in the PG in larvae containing *scrib wts* wing disc tumors and upon overexpression of Upd in the PG (Fig. 6A, quantified in B), whereas levels in tracheae associated with the ring gland were not affected. Apt levels in the PG, in addition, were reduced upon *Stat92E* knockdown in unchallenged larvae, and also in larvae with tumors upon dominant-negative Dome^ΔCYT^ expression (Fig. 6A,B). All these results indicate that Apt expression is under positive JAK/STAT regulation in the PG.

To test the effect of Apt upregulation, we overexpressed Apt in the PG. Apt overexpression under control of *phm-GAL4* produced larvae that did not progress beyond the second-instar (L2) stage. However, when Apt overexpression was restricted to the L2 and L3 stages by using the GAL80^ts^ system, we observed hypertrophied PGs with enlarged nuclei (Fig. 6C,D) and inhibition of the larva–pupa transition (Fig. 6E), similar to the effect of JAK/STAT hyperactivation. Apt knockdown, in contrast, produced reduction of average PG size and pupation time compared to controls (Fig. 6D,F). Furthermore, Apt knockdown in the PG partially rescued the pupation delay induced by *scrib wts* wing disc tumors (Fig. 6G). All these results show that Apt is a target mediating the effect of JAK/STAT signaling both during normal development and in response to tumors.

**Fig. 7.**
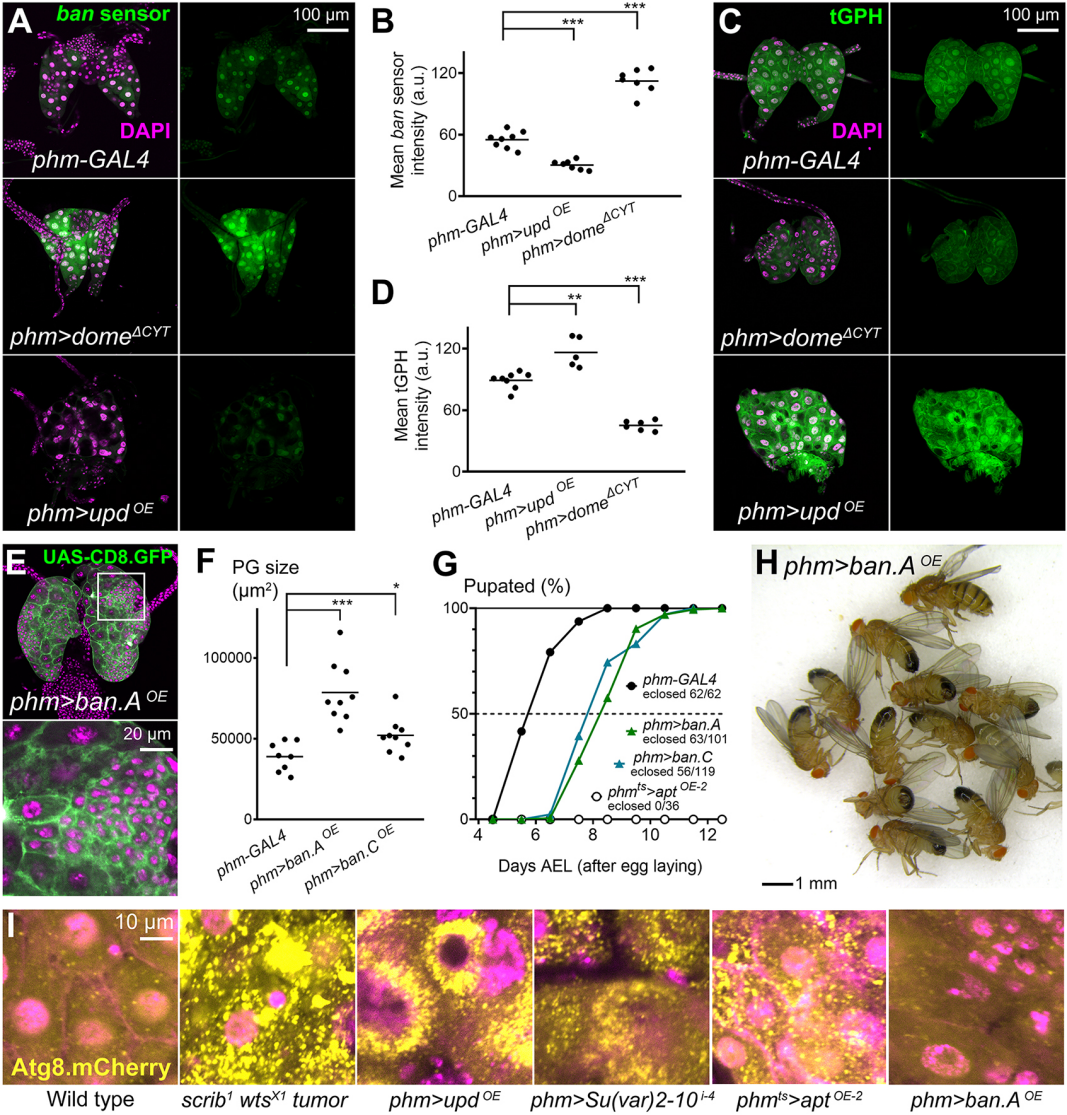
**JAK/STAT targets Apt, and *bantam* (*ban*) differentially affect PG growth and autophagy.** (A) Expression of *ban* sensor (green, separate channel in right column), an anti-reporter for expression of miRNA *ban*, in the PG of control *phm-GAL4* wL3 larvae (upper), *phm>dome^ΔCYT^* wL3 larvae (middle), and giant *phm>upd^OE^* larvae (lower). Higher anti-reporter signal in *phm>dome^ΔCYT^* indicates lower *ban* expression. Nuclei stained with DAPI (magenta). (B) Quantification of *ban* sensor intensity in PG of control *phm-GAL4* wL3 larvae, *phm>dome^ΔCYT^* wL3 larvae and giant *phm>upd^OE^* larvae. Each dot plots mean GFP intensity in the PG measured in images like those in A. Horizontal lines represent mean values. Significance of differences in Mann–Whitney tests is reported. (C) Expression of InR/Pi3K activity reporter tGPH (green, separate channel in right column) in the PG of control *phm-GAL4* wL3 larvae (upper), *phm>dome^ΔCYT^* wL3 larvae (middle) and giant *phm>upd^OE^* larvae (lower). Nuclei stained with DAPI (magenta). (D) Quantification of tGPH intensity in PG of control *phm-GAL4* wL3 larvae, *phm>dome^ΔCYT^* wL3 larvae and giant *phm>upd^OE^* larvae. Each dot plots mean GFP intensity in the PG measured in images like those in C. Horizontal lines represent mean values. Significance of differences in Mann–Whitney tests is reported. (E) Ring gland from a wL3 larva overexpressing *ban* in the PG under *phm-GAL4* control. *phm-GAL4*-driven CD8.GFP in green. Nuclei stained with DAPI (magenta). Higher-magnification inset (white square) in lower panel. (F) Quantification of PG size in control *phm-GAL4* wL3 larvae and larvae overexpressing *ban* under *phm-GAL4* control. Each dot plots PG size measured from images like those in E. Horizontal lines represent mean values. Significance of unpaired two-tailed *t*-tests with Welch's correction are reported. (G) Pupation time in *phm-GAL4* control, *phm>ban^OE^* and *phm^ts^>apt^OE^* animals. Dot-connecting lines plot the accumulated percentage of pupated larvae over time. (H) Despite metamorphosis delay, *phm>ban^OE^* animals reach adulthood, unlike *phm^ts^>apt^OE^* animals. (I) Autophagy marker Atg8.mCherry (yellow) in the PG of wL3 larvae from the indicated genotypes. Nuclei stained with DAPI (magenta). ***P<0.001, **P<0.01 and *P<0.05.

A recent study published while the manuscript was in preparation found that the microRNA *ban* was upregulated downstream of JAK/STAT signaling in the PG in response to tumors (Romão et al., 2021). To test the ability of JAK/STAT to regulate *ban* expression, we used the *ban* sensor anti-reporter (Brennecke et al., 2003). We found elevated *ban* expression in PGs overexpressing Upd and, conversely, reduced *ban* expression upon Dome^ΔCYT^ expression (Fig. 7A,B; see also Fig. S3A for a time course of *ban* sensor during larval development). Importantly, the Dome^ΔCYT^ result shows that JAK/STAT signaling positively regulates *ban* expression during normal PG development (intrinsic signaling) and not just in a damage response context (extrinsic signaling). Insulin receptor (InR) signaling has been reported to downregulate *ban* expression in the PG (Boulan et al., 2013). To assess the possibility that JAK/STAT induced *ban* expression through an effect on upstream InR signaling, we used the tGPH InR/Pi3K activity reporter (Britton et al., 2002). In this way, we observed that Upd expression upregulated InR/Pi3K signaling instead of downregulating it, whereas Dome^ΔCYT^ decreased InR/Pi3K signaling (Fig. 7C,D; see also Fig. S3B for a time course of the tGPH reporter during larval development). These results suggest that JAK/STAT signaling downregulates *ban* expression independent of its possible effects on InR/Pi3K signaling.

**Fig. 8.**
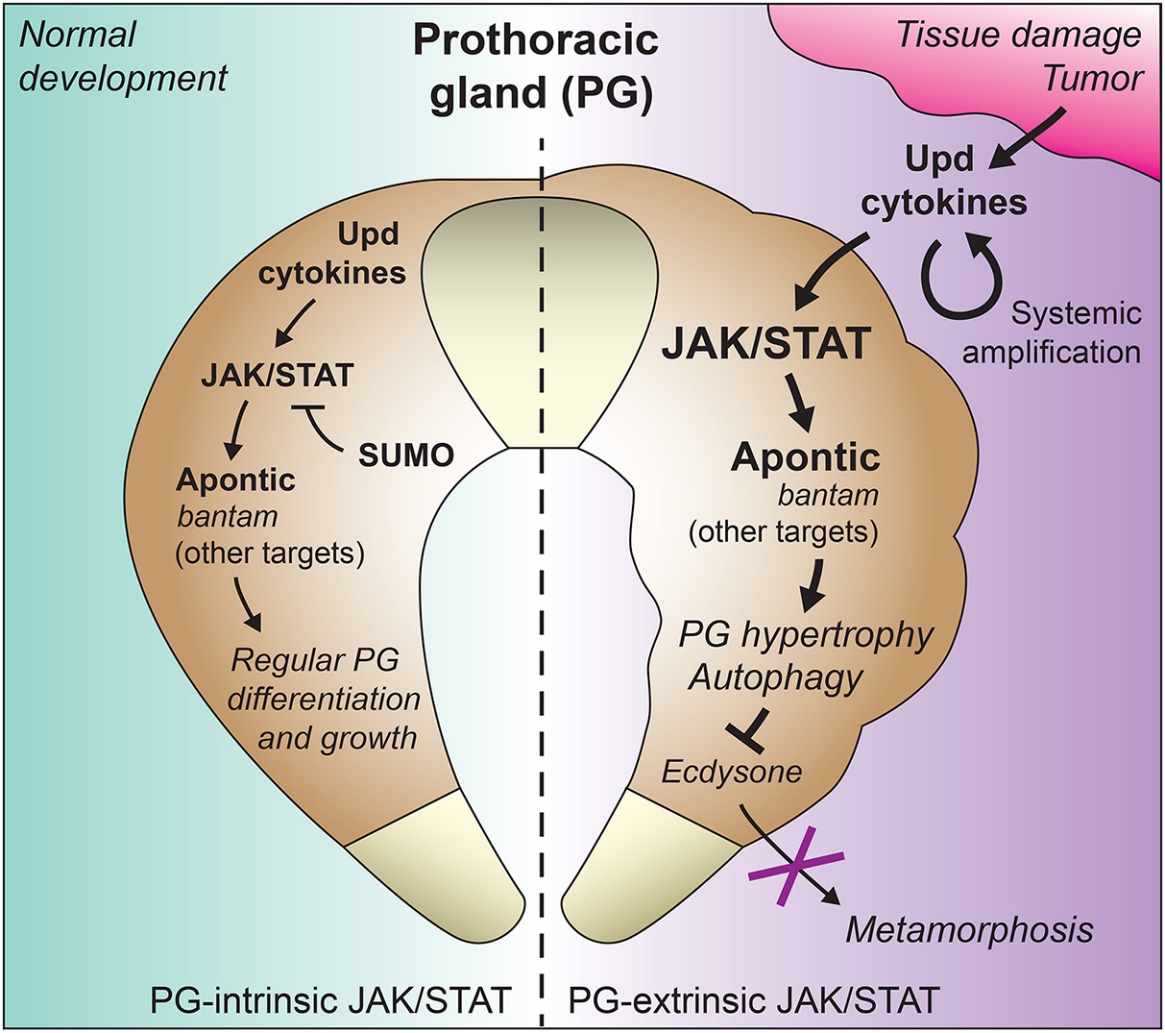
**Intrinsic and extrinsic regulation of PG growth and developmental timing by JAK/STAT.** Schematic model of the function of JAK/STAT signaling in the PG. Intrinsic expression of Upd3 in the PG activates basal levels of JAK/STAT activity in an autocrine way. Basal JAK/STAT activity is required for correct development of the PG, its loss reducing PG size and slightly advancing the onset of metamorphosis (larva–pupa transition). Levels of JAK/STAT activity are controlled through SUMOylation, involving SUMO E3 ligase Su(var)2-10 (PIAS), thus preventing excessive pathway activation. In conditions producing tumors and tissue damage, Upd cytokines secreted from the damaged tissues and amplified by other tissues (Pastor-Pareja et al., 2008) activate JAK/STAT signaling in the PG, which increases expression of JAK/STAT targets Apontic and *bantam*, and differentially contributes to strong autophagy induction and inhibition of metamorphosis.

To further characterize the effects of *ban* upregulation in the PG, we overexpressed *ban* under control of *phm-GAL4* in the PG and found that, like Apt expression, *ban* could also induce PG overgrowth (Fig. 7E,F). However, contrary to Apt expression, *ban*-overexpressing PGs contained regions with large numbers of smaller cells. Also in contrast with Apt overexpression, flies overexpressing *ban* in the PG were capable of pupating and producing adults, whereas Apt-overexpressing flies did not pupate (Fig. 7G,H). Finally, we found that the PG in larvae with *scrib wts* wing disc tumors, similar to those with Upd overexpression, *Su(var)2-10* knockdown and Apt overexpression, presented high levels of autophagy induction, as evidenced by the formation of numerous large Atg.mCherry-positive vesicles in the cytoplasm, consistent with the vacuolation and varying degrees of tissue degeneration in these PGs (Fig. 7I). This is in contrast to what is observed in the *ban*-overexpressing PG, where no such levels of autophagy induction were observed (Fig. 7I). Furthermore, Upd-overexpressing PGs were already highly autophagic before pupation delay onset, in contrast with PGs in which we knocked down expression of *ecdysoneless* (*ecd*), a control condition causing ecdysone deficiency and preventing pupation as well (Fig. S4). This result shows that autophagy upon JAK/STAT activation is not a secondary effect of metamorphosis delay. Our results, in summary, indicate that different JAK/STAT targets, such as Apt and *ban*, may contribute to different aspects of the response to tumors and tissue damage (Fig. 8), leaving for future studies a thorough analysis of these targets, their effects on PG development and their integration with other signals regulating developmental timing.

## DISCUSSION

Our findings show that JAK/STAT signaling is active in the PG at basal levels during normal development. Another study examined JAK/STAT activity in the PG and found expression of the same 10xSTAT-GFP reporter we used (Pan and O'Connor, 2021). We found, in addition, that pupation is slightly advanced and the size of the ring gland is smaller in multiple conditions of JAK/STAT signaling reduction, including knockdown in PG cells of *upd3*, which a GAL4 expression reporter and quantitative reverse transcription PCR (qRT-PCR) show is intrinsically expressed in the ring gland. Similarly, advanced pupation has been reported upon loss of Krüppel homolog 1 (Kr-h1), encoding a transcription factor that represses transcription of steroidogenic enzymes downstream of Juvenile hormone (JH) signaling (Liu et al., 2018; Zhang et al., 2018). Kr-h1 and the JH pathway, thus, seem reasonable candidates for future investigation into the mechanisms by which JAK/STAT activity regulates pupation time.

As for the effects of JAK/STAT reduction on PG size, a developmental time course of reporters for JAK/STAT signaling, *upd3* and *dome* expression suggests that JAK/STAT is active in the PG during all three stages of larval development. Furthermore, JAK/STAT signaling is known to be active in PG cells during embryonic development, when it is involved in the specification of the tracheal primordium that gives rise to the PG and corpus allatum (Sanchez-Higueras et al., 2014). Our results may therefore reveal a continued role of JAK/STAT in the development, growth and correct differentiation of the larval PG.

Our manipulations of JAK/STAT activity changed the size and ploidy of PG cells, but not cell numbers. Despite this, we consider it possible that JAK/STAT regulates growth in general, rather than specifically endoreplication. This is because JAK/STAT seems active before, during and after the switch to endoreplication of PG cells, happening between the L1 and L2 stages. Also in agreement with a growth-stimulating role is the ability of JAK/STAT to upregulate *ban* miRNA expression and Pi3K signaling, potent inducers of proliferation and growth in this and other contexts.

Consistent as well with a role of JAK/STAT in ring gland development, hyperactivation of the pathway caused PG overgrowth and metamorphosis delay, contrary effects to those of loss-of-function conditions. We found in the literature that loss of *Sumo*, encoding fly SUMO, caused a strikingly similar PG hypertrophy phenotype (Talamillo et al., 2008). Our results, showing that *Stat92E* knockdown suppresses the effect of SUMO E3 ligase Su(var)2-10 knockdown, indicate that hyperactivation of JAK/STAT signaling is responsible for this phenotype. This suppression result, in fact, offers strong additional support for a role of JAK/STAT in normal PG development. This is because the human Su(var)2-10 ortholog PIAS has been shown to specifically bind and inhibit only active phosphorylated dimeric STAT, not monomeric STAT (Liao et al., 2000), and thus JAK/STAT hyperactivation in this condition strongly implies a pre-existing level of JAK/STAT activity.

Similar to JAK/STAT hyperactivation, PG overgrowth and ploidy increase are observed in conditions hyperactivating insulin/Ras/Pi3K/Tor signaling (Caldwell et al., 2005; Colombani et al., 2005; Mirth et al., 2005; Ohhara et al., 2017). However, those conditions result in advanced pupation instead. This has been interpreted as revealing a mechanism by which PG size resulting from insulin-driven PG growth mirrors organismal growth to trigger metamorphosis in well-fed animals. PG overgrowth resulting from JAK/STAT activation, in stark contrast, inhibits transcription of enzymes in the ecdysone synthesis pathway and results in absent or delayed metamorphosis. Also in contrast, JAK/STAT-induced PG hypertrophy leads to an aberrant tissue in which vacuolation, autophagy and large differences in cell size are observed. Our results, therefore, highlight that a simple equivalence between large PG size and advanced pupation cannot be taken for granted. Instead, specific aspects of the PG response to insulin different from mere computation of cell size or ploidy must be at play in determining the time of metamorphosis.

In addition to its developmental role, JAK/STAT signaling in the PG is extrinsically activated in response to tumors and tissue damage. Strong local upregulation of all three Upd cytokines has been observed in tumors (Wu et al., 2010), while amplification through systemic upregulation has been observed for Upd3 at least (Pastor-Pareja et al., 2008). Although not investigated here, our results predict that JAK/STAT activation by infection may regulate developmental timing as well, given the involvement of Upd cytokines in the immune response to pathogens (Agaisse and Perrimon, 2004). A previous study postulated that JAK/STAT signaling influences developmental timing by enhancing local expression of Dilp8 in wounded tissue (Katsuyama et al., 2015). Our results show that Upd cytokines, in addition, have a strong unmediated effect in the PG, where they activate JAK/STAT signaling and influence the larva–pupa transition directly. In further support of this, partial rescue of tumor-induced pupation delay by expressing a dominant-negative version of the JAK/STAT receptor Dome in the PG clearly shows that JAK/STAT acts directly in the PG without mediation of Dilp8. Pupation delays induced by tissue damage are only partially rescued in null mutants for *Lgr3* (Garelli et al., 2015), encoding the specific receptor of Dilp8, which suggests that JAK/STAT, Dilp8 and perhaps other signals may be responsible for damage-induced pupation delay in parallel.

A number of pathway ligands acting in tissues in typical developmental patterning and differentiation/growth capacities have been shown to regulate ecdysone production by the PG. Such is the case of Hh, released from enterocytes under starvation conditions (Rodenfels et al., 2014). The TGFβ homolog Dpp, normally escaping from imaginal discs to the hemolymph (Ma et al., 2017), has been shown to downregulate ecdysone production by increasing nuclear Foxo localization and *ban* expression, suggesting that disc-derived Dpp functions as a signal that conveys organismal growth status to the endocrine system (Setiawan et al., 2018). Because Upd cytokines are produced during normal development in multiple organs, including imaginal discs, it is possible that PG-extrinsic Upd expression also serves a coordination role in normal development similar to Dpp. More recently, EGF ligands Spitz and Vein expressed intrinsically in the PG have been shown to regulate pupation as well, in this case stimulating ecdysone production (Cruz et al., 2020). This raises the converse question of whether EGF produced by other tissues can influence timing of the larva–pupa transition. Altogether, these studies paint a scenario in which the distinction between short-range developmental pathway ligands, long-range inter-organ communication hormones and immune response cytokines becomes increasingly blurred.

While we were finishing our study, the group of Marco Milán reported that Upd3 signals from tumors to the PG to delay metamorphosis (Romão et al., 2021), consistent with our findings. According to that study, JAK/STAT inhibits pupation by inducing expression of the *ban* miRNA. Besides demonstrating a JAK/STAT role in normal PG development, our study confirms the ability of Upd cytokines from damaged tissue to delay pupation through *ban* upregulation while, in addition, showing that Apt is a critical target of JAK/STAT signaling in the PG. The effects of Apt expression alone, indeed, closely resembled those of JAK/STAT hyperactivation achieved through Upd overexpression or *Su(var)2-10* knockdown, including tissue hypertrophy and autophagy. The response to JAK/STAT in the PG, therefore, is likely to involve multiple targets. Future studies should clarify the targets and mechanisms by which JAK/STAT signaling regulates developmental timing by the PG.

## MATERIALS AND METHODS

### *Drosophila* strains and genetics

Standard fly husbandry techniques and genetic methodologies were used to assess segregation of transgenes in the progeny of crosses, construct intermediate strains and obtain flies of the desired genotypes for each experiment (Roote and Prokop, 2013). The GAL4-UAS binary expression system (Brand and Perrimon, 1993) was used to drive expression of UAS transgenes under control of GAL4 drivers *phm-GAL4* (PG) and *Cg-GAL4* (fat body). Flies were reared at 25°C on standard fly medium in all experiments, except for *apt* overexpression experiments using the GAL80^ts^ thermosensitive GAL4 repressor (McGuire et al., 2003), in which crosses to obtain *phm^ts^>apt^OE^* animals were left for 3 days at 18°C to avoid early lethality before transferring to 30°C. Sex of experimental animals was not determined except for *hop^25^* (X-linked mutation), where only males can be assessed. Genotypes of flies in all experiments are detailed in Table S1. Original fly strains used in this study are listed in Table S2.

### Imaging and image analysis

Ring glands and wing discs were pre-dissected in PBS by turning larvae inside out with fine-tip forceps, fixed in PBS containing 4% paraformaldehyde (PFA; Sinopharm Chemical Reagent, cat #80096692), washed in PBS (3×10 min), dissected from the carcass and mounted on a glass slide with a drop of DAPI-Vectashield (Vector Laboratories, cat #H-1200). Confocal images were acquired with a Zeiss LSM780 confocal microscope equipped with Plan-Apochromat 20×/0.8 NA objective (ring gland images) and an EC Plan-Neofluar 10×/0.3 NA objective (wing disc images). Images of larvae, pupae and adults were taken with a Leica M125 stereomicroscope, except for the image in Fig. 4A, taken with a Leica MZ10F stereomicroscope.

For quantification of 10xSTAT-GFP, *ban* sensor and tGPH intensity in the PG, the polygon selection in ImageJ-FIJI software was used to outline the area occupied by the PG in confocal images of ring glands, and mean 10xSTAT-GFP intensity (total fluorescence intensity divided by area) was measured inside. At least five ring glands were analyzed in each condition.

For PG size quantification, the polygon selection in ImageJ-FIJI software was used to measure the area occupied by the PG in confocal images of ring glands. At least five ring glands were analyzed for each condition (each point represents the PG size in one ring gland).

For PG nuclear counts, nuclei were manually counted using the Multiple points tool in ImageJ-FIJI software. These counts were conducted on confocal *z*-stack images of DAPI and CD8.GFP (expressed under PG-specific *phm-GAL4* control). At least 18 ring glands were analyzed per genotype.

For ploidy estimation, ring glands from wL3 larvae were dissected, fixed and mounted in DAPI-Vectashield (Vector Laboratories). Then, *z*-stacks of images of DAPI and *phm-GAL4*-driven CD8.GFP were acquired in a Zeiss LSM780 confocal microscope equipped with a Plan-Apochromat 63× oil objective. On those stacks, the volume of PG nuclei was delimited and labeled with the Surface function in Imaris 9.3.1 software (Bitplane), and total DAPI fluorescence inside the nucleus recorded. Ploidy was calculated with reference to the average value in measurements of DAPI fluorescence conducted in the same way in diploid (2C) blood cells.

For quantification of anti-Apt signal, the polygon selection in ImageJ-FIJI software was used to outline the PG nuclei in confocal images of stained ring glands, and mean intensity (total fluorescence intensity divided by area) was measured inside. More than 120 PG nuclei from at least eight ring glands were analyzed for each genotype.

### Immunohistochemistry

Anti-Apt stainings were performed following standard procedures for imaginal discs. Briefly, wL3 larvae were pre-dissected in PBS by turning them inside out with fine-tip forceps, fixed in 4% PFA for 15 min, washed in PBS (3×15 min), blocked in PBT-BSA [PBS containing 0.1% Triton X-100 detergent, 1% bovine serum albumin (BSA) and 250 mM NaCl], incubated overnight with primary anti-Apt (1:1000) in PBT-BSA at 4°C, washed in PBT-BSA (3×20 min), incubated for 2 h with anti-rabbit IgG conjugated to Alexa Fluor-555 (1:200; Life Technologies) in PBT-BSA at room temperature, and washed in PBT-BSA (3×10 min) and PBS (3×10 min). Stained ring glands were finally dissected and mounted on a slide with a drop of DAPI-Vectashield (Vector Laboratories).

### Pupation timing

Fresh virgin female and male flies were kept together in bottles for 3 days for mating and then allowed to lay eggs onto grape juice agar plates for 2 h at 25°C. The next day, newly hatched L1 larvae were picked at 1 h intervals and transferred to standard medium vials on which the top layer of food had been ground with forceps. Twenty to 40 larvae were deposited in each vial. The number of pupated animals was counted at 2-6 h intervals. For the experiments in Fig. 5F and Fig. 6G, egg laying lasted 12 h instead and pupation time was computed in days after egg hatching (±6 h). For the experiment in Fig. 7G, egg laying took place during 1 day in regular food vials and pupation time was computed in days after egg laying (±0.5 days).

### qRT-PCR

Total RNA was extracted from cephalic complexes containing the ring gland using TRIzol reagent (ThermoFisher Scientific), treated with DNase (Promega) and used as a template for cDNA synthesis using iScript™ cDNA Synthesis Kits (Bio-Rad). RT-PCR reactions were performed using SYBR Green Supermix (Bio-Rad) in a CFX96 Real-Time PCR system (Bio-Rad). Expression values were normalized to *RpL23* transcript levels. Fold change with respect to the wild-type control was calculated with Bio-Rad CFX Manager 3.1 software. Three separate biological replicates were performed for each experiment, each with three technical replicates. Primers used were as follows: *RpL23*-F, 5′-GACAACACCGGAGCCAAGAACC-3′; *RpL23*-R, 5′-GTTTGCGCTGCCGAATAACCAC-3′; *phm*-F, 5′-GGATTTCTTTCGGCGCGATGTG-3′; *phm*-R, 5′-TGCCTCAGTATCGAAAAGCCGT-3′; *dib*-F, 5′-TGCCCTCAATCCCTATCTGGTC-3′; *dib*-R, 5′-ACAGGGTCTTCACACCCATCTC-3′; *sad*-F, 5′-CCGCATTCAGCAGTCAGTGG-3′; *sad*-R, 5′-ACCTGCCGTGTACAAGGAGAG-3′; *nvd*-F, 5′-GGAAGCGTTGCTGACGACTGTG-3′; *nvd*-R, 5′-TAAAGCCGTCCACTTCCTGCGA-3′; *upd2*-F, 5′-AGTGCGGTGAAGCTAAAGACTTG-3′; *upd2*-R, 5′-GCCCGTCCCAGATATGAGAA-3′; *upd3*-F, 5′-TGCCCCGTCTGAATCTCACT-3′; *upd3*-R, 5′-GTGAAGGCGCCCACGTAA-3′.

### Wounding assay

Wounding of the larval epidermis was performed on mid-L3 larvae by puncturing at once dorsal and ventral epidermis near the posterior end of the larva with fine-tip forceps. Wounding operations were performed on Sylgard plates on clean, dried larvae, avoiding damage to the gut or other internal organs. Operated larvae were kept on a dry slide for 5 min to allow coagulation at the wound and prevent bleeding. Operated larvae were finally transferred to standard medium vials on which the top layer of food had been ground with forceps.

### Heating assay

Newly hatched L1 larvae were picked at 1 h intervals and transferred to standard food vials after 4 h egg laying in agar juice plates, placing 20 to 40 animals per vial. Four days after egg hatching, mid-L3 larvae were picked and transferred into 3.5-cm plastic dishes filled with ground standard food for heat treatment. The dishes were sealed with parafilm and placed for 2.5 h in a water bath at 25°C (control) or 39°C. After heating, larvae were kept at room temperature for 15 min and then placed back into standard food vials in which the top layer of food had been ground with forceps. Pupation time after egg hatching was recorded.

### 20E treatment

To make 20E working solutions (2.7 mM), we prepared 43.35 mM stocks of 20E (Abcam) in dimethyl sulfoxide and diluted them in PBS. *phm>upd^OE^* L3 larvae 80-96 h after egg hatching were washed, divided into two groups and placed into two different 3.5-cm plastic dishes: one filled with 1 ml 20E working solution and the other with PBS as control. Plates were maintained at 25°C, and the number of pupae was recorded after 4 days.

### Quantification and statistical analysis

Statistical analysis and graphical representations were performed with GraphPad Prism software. Horizontal lines in all graphs represent average values (means). For statistical comparisons, unpaired two-tailed Student's *t*-tests were conducted when data passed both D'Agostino-Pearson normality tests and *F*-tests for equal variance. Unpaired two-tailed *t*-tests with Welch's correction were used when data passed D'Agostino-Pearson normality tests, but not *F*-tests for equal variance. Finally, non-parametric Mann–Whitney tests were used when data did not pass D'Agostino-Pearson normality tests. *P*<0.05 was considered significant.

## Supplementary Material

Supplementary information

## References

[DMM049160C1] Agaisse, H. and Perrimon, N. (2004). The roles of JAK/STAT signaling in *Drosophila* immune responses. *Immunol. Rev.* 198, 72-82. 10.1111/j.0105-2896.2004.0133.x 15199955

[DMM049160C2] Agaisse, H., Petersen, U.-M., Boutros, M., Mathey-Prevot, B. and Perrimon, N. (2003). Signaling role of hemocytes in *Drosophila* JAK/STAT-dependent response to septic injury. *Dev. Cell* 5, 441-450. 10.1016/S1534-5807(03)00244-2 12967563

[DMM049160C3] Akai, N., Ohsawa, S., Sando, Y. and Igaki, T. (2021). Epithelial cell-turnover ensures robust coordination of tissue growth in *Drosophila* ribosomal protein mutants. *PLoS Genet.* 17, e1009300. 10.1371/journal.pgen.1009300 33507966 PMC7842893

[DMM049160C4] Arbouzova, N. I. and Zeidler, M. P. (2006). JAK/STAT signalling in *Drosophila*: insights into conserved regulatory and cellular functions. *Development* 133, 2605-2616. 10.1242/dev.02411 16794031

[DMM049160C5] Bach, E. A., Vincent, S., Zeidler, M. P. and Perrimon, N. (2003). A sensitized genetic screen to identify novel regulators and components of the Drosophila Janus kinase/signal transducer and activator of transcription pathway. *Genetics* 165, 1149-1166. 10.1093/genetics/165.3.1149 14668372 PMC1462825

[DMM049160C6] Bach, E. A., Ekas, L. A., Ayala-Camargo, A., Flaherty, M. S., Lee, H., Perrimon, N. and Baeg, G.-H. (2007). GFP reporters detect the activation of the *Drosophila* JAK/STAT pathway *in vivo*. *Gene Expr. Patterns* 7, 323-331. 10.1016/j.modgep.2006.08.003 17008134

[DMM049160C7] Barredo, C. G., Gil-Marti, B., Deveci, D., Romero, N. M. and Martin, F. A. (2021). Timing the juvenile-adult neurohormonal transition: functions and evolution. *Front. Endocrinol.* 11, 602285. 10.3389/fendo.2020.602285 PMC7909313 33643219

[DMM049160C8] Beccari, S., Teixeira, L. and Rørth, P. (2002). The JAK/STAT pathway is required for border cell migration during *Drosophila* oogenesis. *Mech. Dev.* 111, 115-123. 10.1016/S0925-4773(01)00615-3 11804783

[DMM049160C9] Betz, A., Lampen, N., Martinek, S., Young, M. W. and Darnell, J. E. Jr. (2001). A *Drosophila* PIAS homologue negatively regulates *stat92E*. *Proc. Natl. Acad. Sci. USA* 98, 9563-9568. 10.1073/pnas.171302098 11504941 PMC55492

[DMM049160C10] Binari, R. and Perrimon, N. (1994). Stripe-specific regulation of pair-rule genes by hopscotch, a putative Jak family tyrosine kinase in Drosophila. *Genes Dev.* 8, 300-312. 10.1101/gad.8.3.300 8314084

[DMM049160C11] Boulan, L., Martín, D. and Milán, M. (2013). bantam miRNA promotes systemic growth by connecting insulin signaling and ecdysone production. *Curr. Biol.* 23, 473-478. 10.1016/j.cub.2013.01.072 23477723

[DMM049160C12] Brand, A. H. and Perrimon, N. (1993). Targeted gene expression as a means of altering cell fates and generating dominant phenotypes. *Development* 118, 401-415. 10.1242/dev.118.2.401 8223268

[DMM049160C13] Brennecke, J., Hipfner, D. R., Stark, A., Russell, R. B. and Cohen, S. M. (2003). *bantam* encodes a developmentally regulated microRNA that controls cell proliferation and regulates the proapoptotic gene *hid* in *Drosophila*. *Cell* 113, 25-36. 10.1016/S0092-8674(03)00231-9 12679032

[DMM049160C14] Britton, J. S., Lockwood, W. K., Li, L., Cohen, S. M. and Edgar, B. A. (2002). *Drosophila*’s insulin/PI3-kinase pathway coordinates cellular metabolism with nutritional conditions. *Dev. Cell* 2, 239-249. 10.1016/S1534-5807(02)00117-X 11832249

[DMM049160C15] Brown, S., Hu, N. and Hombría, J. C.-G. (2001). Identification of the first invertebrate interleukin JAK/STAT receptor, the *Drosophila* gene *domeless*. *Curr. Biol.* 11, 1700-1705. 10.1016/S0960-9822(01)00524-3 11696329

[DMM049160C16] Bryant, P. J. (1971). Regeneration and duplication following operations *in situ* on the imaginal discs of *Drosophila melanogaster*. *Dev. Biol.* 26, 637-651. 10.1016/0012-1606(71)90146-1 5002603

[DMM049160C17] Caldwell, P. E., Walkiewicz, M. and Stern, M. (2005). Ras activity in the *Drosophila* prothoracic gland regulates body size and developmental rate via ecdysone release. *Curr. Biol.* 15, 1785-1795. 10.1016/j.cub.2005.09.011 16182526

[DMM049160C18] Chen, H.-W., Chen, X., Oh, S.-W., Marinissen, M. J., Gutkind, J. S. and Hou, S. X. (2002). *mom* identifies a receptor for the *Drosophila* JAK/STAT signal transduction pathway and encodes a protein distantly related to the mammalian cytokine receptor family. *Genes Dev.* 16, 388-398. 10.1101/gad.955202 11825879 PMC155335

[DMM049160C19] Colombani, J., Bianchini, L., Layalle, S., Pondeville, E., Dauphin-Villemant, C., Antoniewski, C., Carré, C., Noselli, S. and Léopold, P. (2005). Antagonistic actions of ecdysone and insulins determine final size in *Drosophila*. *Science* 310, 667-670. 10.1126/science.1119432 16179433

[DMM049160C20] Colombani, J., Andersen, D. S. and Léopold, P. (2012). Secreted peptide Dilp8 coordinates *Drosophila* tissue growth with developmental timing. *Science* 336, 582-585. 10.1126/science.1216689 22556251

[DMM049160C21] Cruz, J., Martín, D. and Franch-Marro, X. (2020). Egfr signaling is a major regulator of ecdysone biosynthesis in the Drosophila prothoracic gland. *Curr. Biol.* 30, 1547-1554.e4. 10.1016/j.cub.2020.01.092 32220314

[DMM049160C22] Díaz-García, S. and Baonza, A. (2013). Pattern reorganization occurs independently of cell division during *Drosophila* wing disc regeneration in situ. *Proc. Natl. Acad. Sci. USA* 110, 13032-13037. 10.1073/pnas.1220543110 23878228 PMC3740865

[DMM049160C23] Eulenberg, K. G. and Schuh, R. (1997). The *tracheae defective* gene encodes a bZIP protein that controls tracheal cell movement during *Drosophila* embryogenesis. *EMBO J.* 16, 7156-7165. 10.1093/emboj/16.23.7156 9384592 PMC1170316

[DMM049160C24] Garelli, A., Gontijo, A. M., Miguela, V., Caparros, E. and Dominguez, M. (2012). Imaginal discs secrete insulin-like peptide 8 to mediate plasticity of growth and maturation. *Science* 336, 579-582. 10.1126/science.1216735 22556250

[DMM049160C25] Garelli, A., Heredia, F., Casimiro, A. P., Macedo, A., Nunes, C., Garcez, M., Dias, A. R. M., Volonte, Y. A., Uhlmann, T., Caparros, E. et al. (2015). Dilp8 requires the neuronal relaxin receptor Lgr3 to couple growth to developmental timing. *Nat. Commun.* 6, 8732. 10.1038/ncomms9732 26510564 PMC4640092

[DMM049160C26] Gilbert, M. M., Weaver, B. K., Gergen, J. P. and Reich, N. C. (2005). A novel functional activator of the Drosophila JAK/STAT pathway, unpaired2, is revealed by an in vivo reporter of pathway activation. *Mech. Dev.* 122, 939-948. 10.1016/j.mod.2005.03.004 15925495

[DMM049160C27] Grönholm, J., Ungureanu, D., Vanhatupa, S., Rämet, M. and Silvennoinen, O. (2010). Sumoylation of Drosophila transcription factor STAT92E. *J. Innate Immunity* 2, 618-624. 10.1159/000318676 20616536

[DMM049160C28] Hackney, J. F., Zolali-Meybodi, O. and Cherbas, P. (2012). Tissue damage disrupts developmental progression and ecdysteroid biosynthesis in *Drosophila*. *PLoS ONE* 7, e49105. 10.1371/journal.pone.0049105 23166607 PMC3496736

[DMM049160C78] Halme, A., Cheng, M. and Hariharan, I. K. (2010). Retinoids regulate a developmental checkpoint for tissue regeneration in *Drosophila*. *Curr. Biol.* 20, 458-463. 10.1016/j.cub.2010.01.038 20189388 PMC2847081

[DMM049160C29] Hariharan, I. K. and Serras, F. (2017). Imaginal disc regeneration takes flight. *Curr. Opinion Cell Biol.* 48, 10-16. 10.1016/j.ceb.2017.03.005 28376317 PMC5591769

[DMM049160C30] Harrison, D. A., McCoon, P. E., Binari, R., Gilman, M. and Perrimon, N. (1998). *Drosophila unpaired* encodes a secreted protein that activates the JAK signaling pathway. *Genes Dev.* 12, 3252-3263. 10.1101/gad.12.20.3252 9784499 PMC317220

[DMM049160C31] Herrera, S. C. and Bach, E. A. (2019). JAK/STAT signaling in stem cells and regeneration: from *Drosophila* to vertebrates. *Development* 146, dev167643. 10.1242/dev.167643 30696713 PMC6361132

[DMM049160C32] Hombría, J. C.-G., Brown, S., Häder, S. and Zeidler, M. P. (2005). Characterisation of Upd2, a *Drosophila* JAK/STAT pathway ligand. *Dev. Biol.* 288, 420-433. 10.1016/j.ydbio.2005.09.040 16277982

[DMM049160C33] Hou, X. S., Melnick, M. B. and Perrimon, N. (1996). *marelle* acts downstream of the Drosophila HOP/JAK kinase and encodes a protein similar to the mammalian STATs. *Cell* 84, 411-419. 10.1016/S0092-8674(00)81286-6 8608595

[DMM049160C34] Katsuyama, T., Comoglio, F., Seimiya, M., Cabuy, E. and Paro, R. (2015). During *Drosophila* disc regeneration, JAK/STAT coordinates cell proliferation with Dilp8-mediated developmental delay. *Proc. Natl. Acad. Sci. USA* 112, E2327-E2336. 10.1073/pnas.1423074112 25902518 PMC4426433

[DMM049160C35] La Fortezza, M., Schenk, M., Cosolo, A., Kolybaba, A., Grass, I. and Classen, A.-K. (2016). JAK/STAT signalling mediates cell survival in response to tissue stress. *Development* 143, 2907-2919. 10.1242/dev.132340 27385008

[DMM049160C36] Lemaitre, B. and Hoffmann, J. (2007). The host defense of *Drosophila melanogaster*. *Annu. Rev. Immunol.* 25, 697-743. 10.1146/annurev.immunol.25.022106.141615 17201680

[DMM049160C37] Liao, J., Fu, Y. and Shuai, K. (2000). Distinct roles of the NH2- and COOH-terminal domains of the protein inhibitor of activated signal transducer and activator of transcription (STAT) 1 (PIAS1) in cytokine-induced PIAS1-Stat1 interaction. *Proc. Natl. Acad. Sci. USA* 97, 5267-5272. 10.1073/pnas.97.10.5267 10805787 PMC25817

[DMM049160C38] Liu, Q.-X., Wang, X.-F., Ikeo, K., Hirose, S., Gehring, W. J. and Gojobori, T. (2014). Evolutionarily conserved transcription factor Apontic controls the G1/S progression by inducing cyclin E during eye development. *Proc. Natl. Acad. Sci. USA* 111, 9497-9502. 10.1073/pnas.1407145111 24979795 PMC4084451

[DMM049160C39] Liu, S., Li, K., Gao, Y., Liu, X., Chen, W., Ge, W., Feng, Q., Palli, S. R. and Li, S. (2018). Antagonistic actions of juvenile hormone and 20-hydroxyecdysone within the ring gland determine developmental transitions in *Drosophila*. *Proc. Natl. Acad. Sci. USA* 115, 139-144. 10.1073/pnas.1716897115 29255055 PMC5776822

[DMM049160C40] Ma, M., Cao, X., Dai, J. and Pastor-Pareja, J. C. (2017). Basement membrane manipulation in *Drosophila* wing discs affects Dpp retention but not growth mechanoregulation. *Dev. Cell* 42, 97-106.e4. 10.1016/j.devcel.2017.06.004 28697337

[DMM049160C41] McGuire, S. E., Le, P. T., Osborn, A. J., Matsumoto, K. and Davis, R. L. (2003). Spatiotemporal rescue of memory dysfunction in *Drosophila*. *Science* 302, 1765-1768. 10.1126/science.1089035 14657498

[DMM049160C42] Menut, L., Vaccari, T., Dionne, H., Hill, J., Wu, G. and Bilder, D. (2007). A mosaic genetic screen for Drosophila neoplastic tumor suppressor genes based on defective pupation. *Genetics* 177, 1667-1677. 10.1534/genetics.107.078360 17947427 PMC2147992

[DMM049160C43] Mirth, C., Truman, J. W. and Riddiford, L. M. (2005). The role of the prothoracic gland in determining critical weight for metamorphosis in *Drosophila melanogaster*. *Curr. Biol.* 15, 1796-1807. 10.1016/j.cub.2005.09.017 16182527

[DMM049160C44] Ohhara, Y., Kobayashi, S. and Yamanaka, N. (2017). Nutrient-dependent endocycling in steroidogenic tissue dictates timing of metamorphosis in *Drosophila melanogaster*. *PLoS Genet.* 13, e1006583. 10.1371/journal.pgen.1006583 28121986 PMC5298324

[DMM049160C45] Osman, D., Buchon, N., Chakrabarti, S., Huang, Y.-T., Su, W.-C., Poidevin, M., Tsai, Y.-C. and Lemaitre, B. (2012). Autocrine and paracrine unpaired signaling regulate intestinal stem cell maintenance and division. *J. Cell Sci.* 125, 5944-5949. 10.1242/jcs.113100 23038775

[DMM049160C46] Pagliarini, R. A. and Xu, T. (2003). A genetic screen in *Drosophila* for metastatic behavior. *Science* 302, 1227-1231. 10.1126/science.1088474 14551319

[DMM049160C47] Pan, X. and O'Connor, M. B. (2021). Coordination among multiple receptor tyrosine kinase signals controls *Drosophila* developmental timing and body size. *Cell Rep.* 36, 109644. 10.1016/j.celrep.2021.109644 34469735 PMC8428980

[DMM049160C48] Pastor-Pareja, J. C. and Xu, T. (2013). Dissecting social cell biology and tumors using *Drosophila* genetics. *Annu. Rev. Genet.* 47, 51-74. 10.1146/annurev-genet-110711-155414 23988119 PMC3970289

[DMM049160C49] Pastor-Pareja, J. C., Wu, M. and Xu, T. (2008). An innate immune response of blood cells to tumors and tissue damage in Drosophila. *Dis. Model. Mech.* 1, 144-154; discussion 153. 10.1242/dmm.000950 19048077 PMC2562178

[DMM049160C50] Perrimon, N. and Mahowald, A. P. (1986). *l(1)hopscotch*, a larval-pupal zygotic lethal with a specific maternal effect on segmentation in *Drosophila*. *Dev. Biol.* 118, 28-41. 10.1016/0012-1606(86)90070-9 3095163

[DMM049160C51] Rajan, A. and Perrimon, N. (2012). *Drosophila* cytokine Unpaired 2 regulates physiological homeostasis by remotely controlling insulin secretion. *Cell* 151, 123-137. 10.1016/j.cell.2012.08.019 23021220 PMC3475207

[DMM049160C52] Rawlings, J. S., Rosler, K. M. and Harrison, D. A. (2004). The JAK/STAT signaling pathway. *J. Cell Sci.* 117, 1281-1283. 10.1242/jcs.00963 15020666

[DMM049160C53] Rodenfels, J., Lavrynenko, O., Ayciriex, S., Sampaio, J. L., Carvalho, M., Shevchenko, A. and Eaton, S. (2014). Production of systemically circulating Hedgehog by the intestine couples nutrition to growth and development. *Genes Dev.* 28, 2636-2651. 10.1101/gad.249763.114 25452274 PMC4248294

[DMM049160C54] Rodrigues, A. B., Zoranovic, T., Ayala-Camargo, A., Grewal, S., Reyes-Robles, T., Krasny, M., Wu, D. C., Johnston, L. A. and Bach, E. A. (2012). Activated STAT regulates growth and induces competitive interactions independently of Myc, Yorkie, Wingless and ribosome biogenesis. *Development* 139, 4051-4061. 10.1242/dev.076760 22992954 PMC3472591

[DMM049160C55] Rodrigues, D., Renaud, Y., VijayRaghavan, K., Waltzer, L. and Inamdar, M. S. (2021). Differential activation of JAK-STAT signaling reveals functional compartmentalization in *Drosophila* blood progenitors. *eLife* 10, e61409. 10.7554/eLife.61409 33594977 PMC7920551

[DMM049160C56] Romão, D., Muzzopappa, M., Barrio, L. and Milán, M. (2021). The Upd3 cytokine couples inflammation to maturation defects in *Drosophila*. *Curr. Biol.* 31, 1780-1787.e6. 10.1016/j.cub.2021.01.080 33609452

[DMM049160C57] Roote, J. and Prokop, A. (2013). How to design a genetic mating scheme: a basic training package for *Drosophila* genetics. *G3* 3, 353-358. 10.1534/g3.112.004820 23390611 PMC3564995

[DMM049160C58] Sanchez-Higueras, C., Sotillos, S. and Castelli-Gair Hombria, J. (2014). Common origin of insect trachea and endocrine organs from a segmentally repeated precursor. *Curr. Biol.* 24, 76-81. 10.1016/j.cub.2013.11.010 24332544

[DMM049160C59] Santabarbara-Ruiz, P., Lopez-Santillan, M., Martinez-Rodriguez, I., Binagui-Casas, A., Perez, L., Milan, M., Corominas, M. and Serras, F. (2015). ROS-induced JNK and p38 signaling is required for unpaired cytokine activation during *Drosophila* regeneration. *PLoS Genet.* 11, e1005595. 10.1371/journal.pgen.1005595 26496642 PMC4619769

[DMM049160C60] Sehnal, F. and Bryant, P. J. (1993). Delayed pupariation in Drosophila imaginal disc overgrowth mutants is associated with reduced ecdysteroid titer. *J. Insect Physiol.* 39, 1051-1059. 10.1016/0022-1910(93)90129-f

[DMM049160C61] Setiawan, L., Pan, X., Woods, A. L., O'Connor, M. B. and Hariharan, I. K. (2018). The BMP2/4 ortholog Dpp can function as an inter-organ signal that regulates developmental timing. *Life Sci. Alliance* 1, e201800216. 10.26508/lsa.201800216 30515478 PMC6243201

[DMM049160C62] Silver, D. L. and Montell, D. J. (2001). Paracrine signaling through the JAK/STAT pathway activates invasive behavior of ovarian epithelial cells in *Drosophila*. *Cell* 107, 831-841. 10.1016/S0092-8674(01)00607-9 11779460

[DMM049160C63] Simpson, P. and Schneiderman, H. A. (1975). Isolation of temperature sensitive mutations blocking clone development in*Drosophila melanogaster*, and the effects of a temperature sensitive cell lethal mutation on pattern formation in imaginal discs. *Wilhelm Roux Arch. Dev. Biol.* 178, 247-275. 10.1007/BF00848432 28304775

[DMM049160C64] Smith-Bolton, R. K., Worley, M. I., Kanda, H. and Hariharan, I. K. (2009). Regenerative growth in *Drosophila* imaginal discs is regulated by Wingless and Myc. *Dev. Cell* 16, 797-809. 10.1016/j.devcel.2009.04.015 19531351 PMC2705171

[DMM049160C65] Starz-Gaiano, M., Melani, M., Wang, X., Meinhardt, H. and Montell, D. J. (2008). Feedback inhibition of JAK/STAT signaling by apontic is required to limit an invasive cell population. *Dev. Cell* 14, 726-738. 10.1016/j.devcel.2008.03.005 18477455

[DMM049160C66] Stieper, B. C., Kupershtok, M., Driscoll, M. V. and Shingleton, A. W. (2008). Imaginal discs regulate developmental timing in *Drosophila melanogaster*. *Dev. Biol.* 321, 18-26. 10.1016/j.ydbio.2008.05.556 18632097

[DMM049160C67] Talamillo, A., Sanchez, J., Cantera, R., Perez, C., Martin, D., Caminero, E. and Barrio, R. (2008). Smt3 is required for *Drosophila melanogaster* metamorphosis. *Development* 135, 1659-1668. 10.1242/dev.020685 18367553

[DMM049160C68] Tennessen, J. M. and Thummel, C. S. (2011). Coordinating growth and maturation - insights from *Drosophila*. *Curr. Biol.* 21, R750-R757. 10.1016/j.cub.2011.06.033 21959165 PMC4353487

[DMM049160C69] Texada, M. J., Koyama, T. and Rewitz, K. (2020). Regulation of body size and growth control. *Genetics* 216, 269-313. 10.1534/genetics.120.303095 33023929 PMC7536854

[DMM049160C70] Truman, J. W. and Riddiford, L. M. (2019). The evolution of insect metamorphosis: a developmental and endocrine view. *Philos. Trans. R. Soc. Lond. B Biol. Sci.* 374, 20190070. 10.1098/rstb.2019.0070 31438820 PMC6711285

[DMM049160C71] Wu, M., Pastor-Pareja, J. C. and Xu, T. (2010). Interaction between *Ras^V12^* and *scribbled* clones induces tumour growth and invasion. *Nature* 463, 545-548. 10.1038/nature08702 20072127 PMC2835536

[DMM049160C72] Yamanaka, N., Rewitz, K. F. and O'Connor, M. B. (2013). Ecdysone control of developmental transitions: lessons from *Drosophila* research. *Annu. Rev. Entomol.* 58, 497-516. 10.1146/annurev-ento-120811-153608 23072462 PMC4060523

[DMM049160C73] Yan, R., Small, S., Desplan, C., Dearolf, C. R. and Darnell, J. E. Jr. (1996). Identification of a *Sta*t gene that functions in Drosophila development. *Cell* 84, 421-430. 10.1016/S0092-8674(00)81287-8 8608596

[DMM049160C74] Yasugi, T., Umetsu, D., Murakami, S., Sato, M. and Tabata, T. (2008). *Drosophila* optic lobe neuroblasts triggered by a wave of proneural gene expression that is negatively regulated by JAK/STAT. *Development* 135, 1471-1480. 10.1242/dev.019117 18339672

[DMM049160C75] Zeidler, M. P. and Bausek, N. (2013). The *Drosophila* JAK-STAT pathway. *JAK-STAT* 2, e25353. 10.4161/jkst.25353 24069564 PMC3772116

[DMM049160C76] Zeidler, M. P., Perrimon, N. and Strutt, D. I. (1999). Polarity determination in the *Drosophila* eye: a novel role for unpaired and JAK/STAT signaling. *Genes Dev.* 13, 1342-1353. 10.1101/gad.13.10.1342 10346822 PMC316719

[DMM049160C77] Zhang, T., Song, W., Li, Z., Qian, W., Wei, L., Yang, Y., Wang, W., Zhou, X., Meng, M., Peng, J. et al. (2018). Krüppel homolog 1 represses insect ecdysone biosynthesis by directly inhibiting the transcription of steroidogenic enzymes. *Proc. Natl. Acad. Sci. USA* 115, 3960-3965. 10.1073/pnas.1800435115 29567866 PMC5899488

