# Supplementary material for: Intrinsic and damage-induced JAK/STAT signaling regulate developmental timing by the Drosophila prothoracic gland
Source: Dis Model Mech. 2022 Jan 26;15(1):dmm049160. doi: 10.1242/dmm.049160 (PMC8807578; doi:10.1242/dmm.049160)
Supplement: Supplementary information [file dmm-15-049160-s1.pdf]

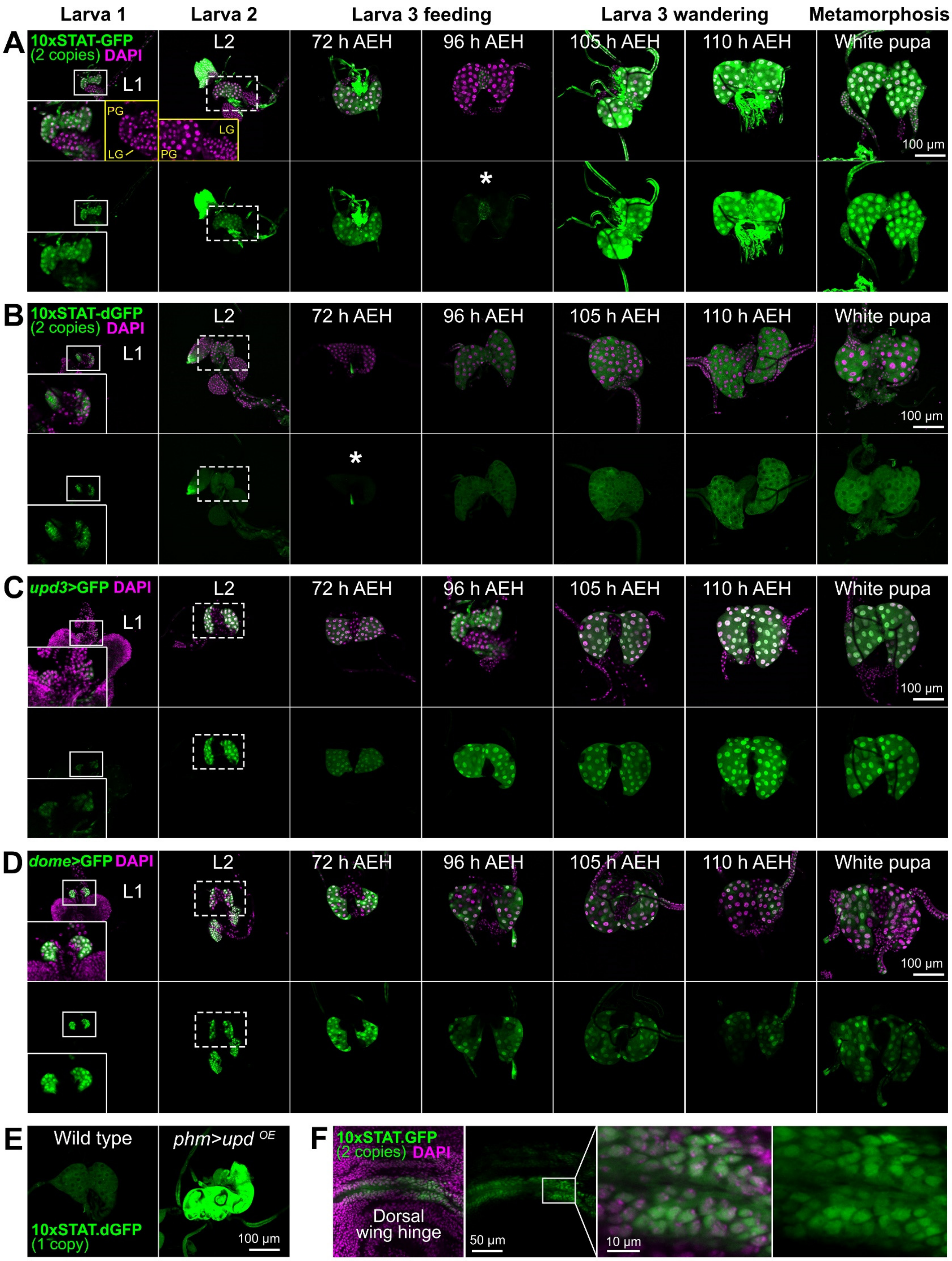

Disease Models & Mechanisms • Supplementary information

Fig. S1. Reporters of JAK/STAT activity, *upd3* and *dome* expression during larval PG development

(A) Expression of JAK/STAT activity reporter 10xSTAT-GFP (enhanced GFP, two copies, green, separate channel in lower subpanels) in the PG during development from L1 to white pupa (0-1 h after metamorphosis onset). Higher magnification insets show the L1 ring gland (white squares). Dotted rectangles highlight L2 ring gland. L3 larvae were dissected at indicated times after egg hatching. Nuclei stained with DAPI (magenta). Notice that nuclear PG size is similar to that of lymph gland (LG) diploid hemocytes in L1, but larger in L2 (yellow insets), indicating that switch to endoreplication in PG cells occurs between these two time points.

(B) Expression of JAK/STAT activity reporter 10xSTAT-dGFP (degenerated GFP, two copies, green, separate channel in lower subpanels) in the PG from L1 to white pupa. Higher magnification insets show the L1 ring gland (white squares). Dotted rectangles highlight L2 ring gland. L3 larvae were dissected at indicated times after egg hatching. Nuclei stained with DAPI (magenta). Notice lower reporter signal at 72 h AEH (asterisk), consistent with lower signal at 96 h AEH for 10xSTAT-GFP once difference in half-life between dGFP and EGFP is taken into account (8 h vs 24 h half-life, respectively).

(C) Ring glands from larvae expressing GFP (green, separate channel in lower subpanels) under control of *upd3-GAL4* from L1 to white pupa. Higher magnification insets show the L1 ring gland (white squares). Dotted rectangles highlight L2 ring gland. L3 larvae were dissected at indicated times after egg hatching. Nuclei stained with DAPI (magenta).

(D) Ring glands from larvae expressing GFP (green, separate channel in lower subpanels) under control of *dome-GAL4* from L1 to white pupa. Higher magnification insets show the L1 ring gland (white squares). Dotted rectangles highlight L2 ring gland. L3 larvae were dissected at indicated times after egg hatching. Nuclei stained with DAPI (magenta).

(E) Expression of JAK/STAT activity reporter 10xSTAT-dGFP (one copy, green) in PG of control *phm-GAL4* wL3 larvae and *phm>upd*^{OE} larvae.

(F) Nuclear GFP localization of 10xSTAT-GFP (two copies, green, separate channel in the second subpanel) in the dorsal hinge of the wing disc. Higher magnification images are shown on the right. Nuclei stained with DAPI (magenta).

Disease Models & Mechanisms • Supplementary information

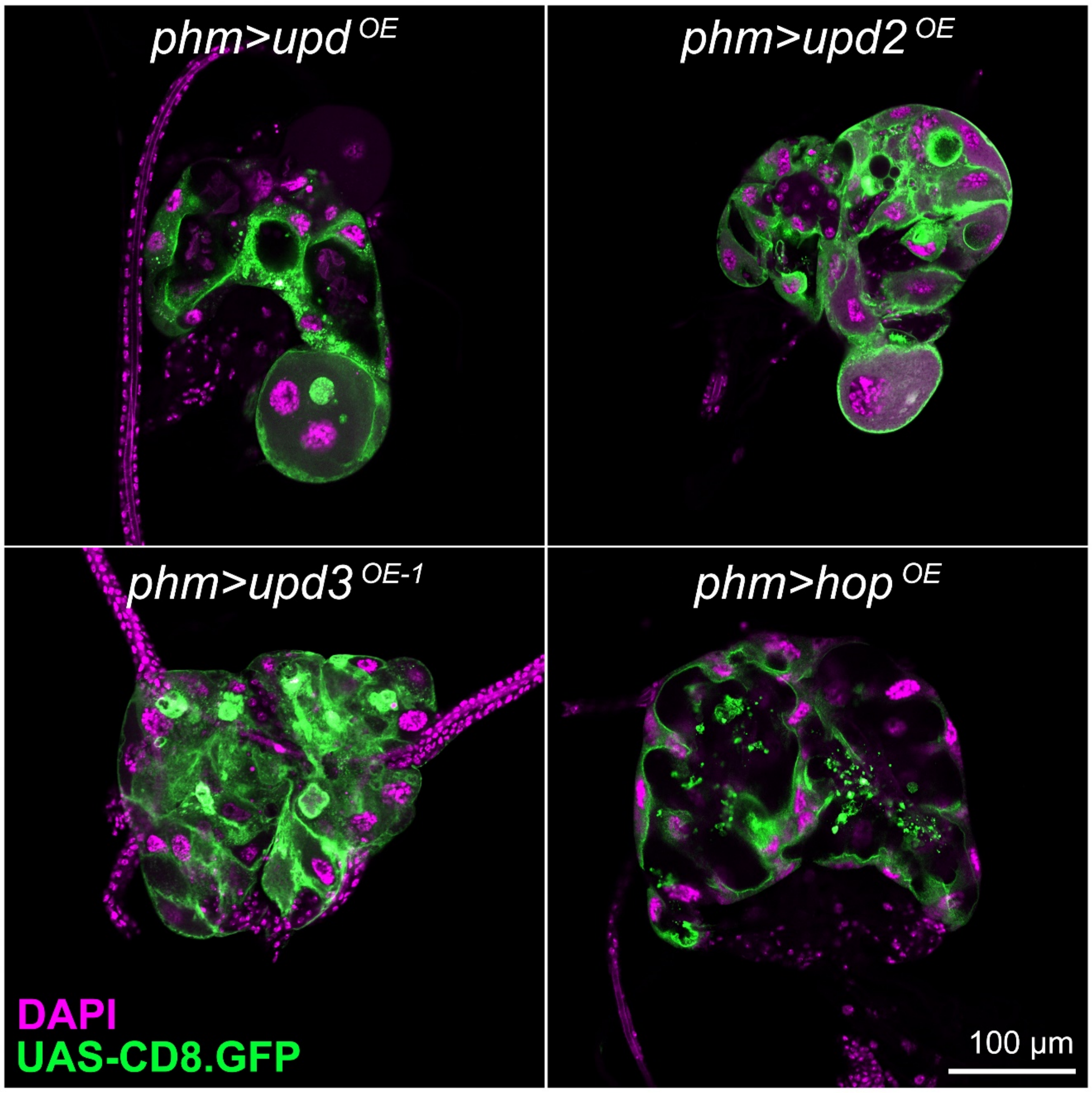

Fig. S2. JAK/STAT hyperactivation induces abnormal PG development

Ring glands from L3 giant larvae overexpressing *upd*, *upd2*, *upd3* and *hop* in the PG under control of *phm-GAL4*. *phm-GAL4*-driven CD8.GFP in green. Nuclei stained with DAPI (magenta). Notice abundant vacuoles and vesicles, as well as variability in cell size and nuclear size.

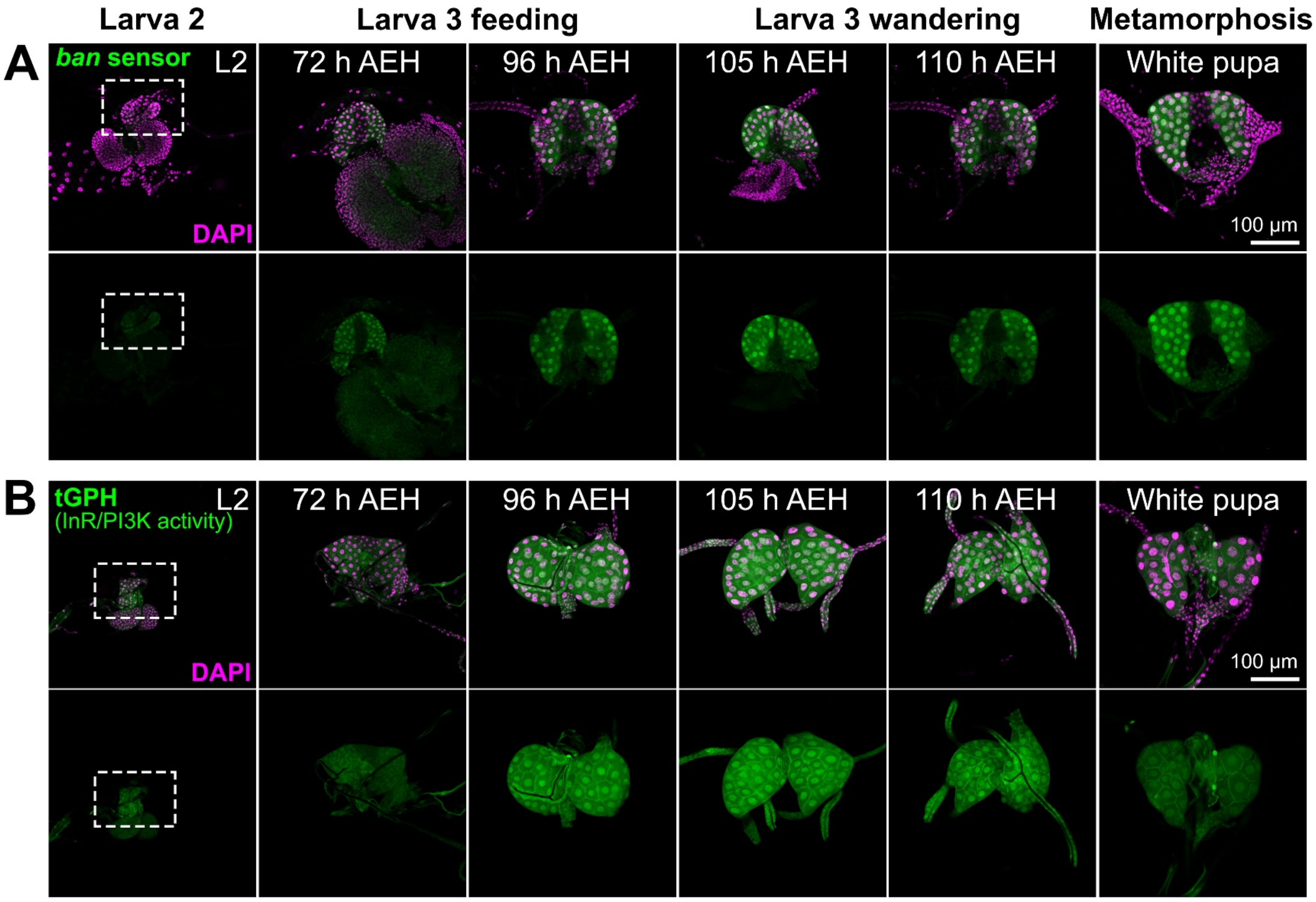

Fig. S3. Expression of *bantam* sensor antireporter and InR/PI3K reporter tGPH during larval PG development

(A) Time course of *bantam* (*ban*) sensor expression (green, separate channel in lower subpanels) in the PG during development from L2 to white pupa. L3 larvae were dissected at indicated times after egg hatching. Dotted rectangles highlight the ring gland of L2 animals. Nuclei stained with DAPI (magenta). Levels of expression of the reporter are anticorrelated to miRNA *ban* expression (Brennecke et al., 2003) and seem constant during L3.

(B) Time course of expression of PI3K signaling reporter tGPH (green, separate channel in lower subpanels) in the PG during development from L2 to white pupa. L3 larvae were dissected at indicated times after egg hatching. Dotted rectangles highlight the ring gland of L2 animals. Nuclei stained with DAPI (magenta).

Disease Models & Mechanisms • Supplementary information

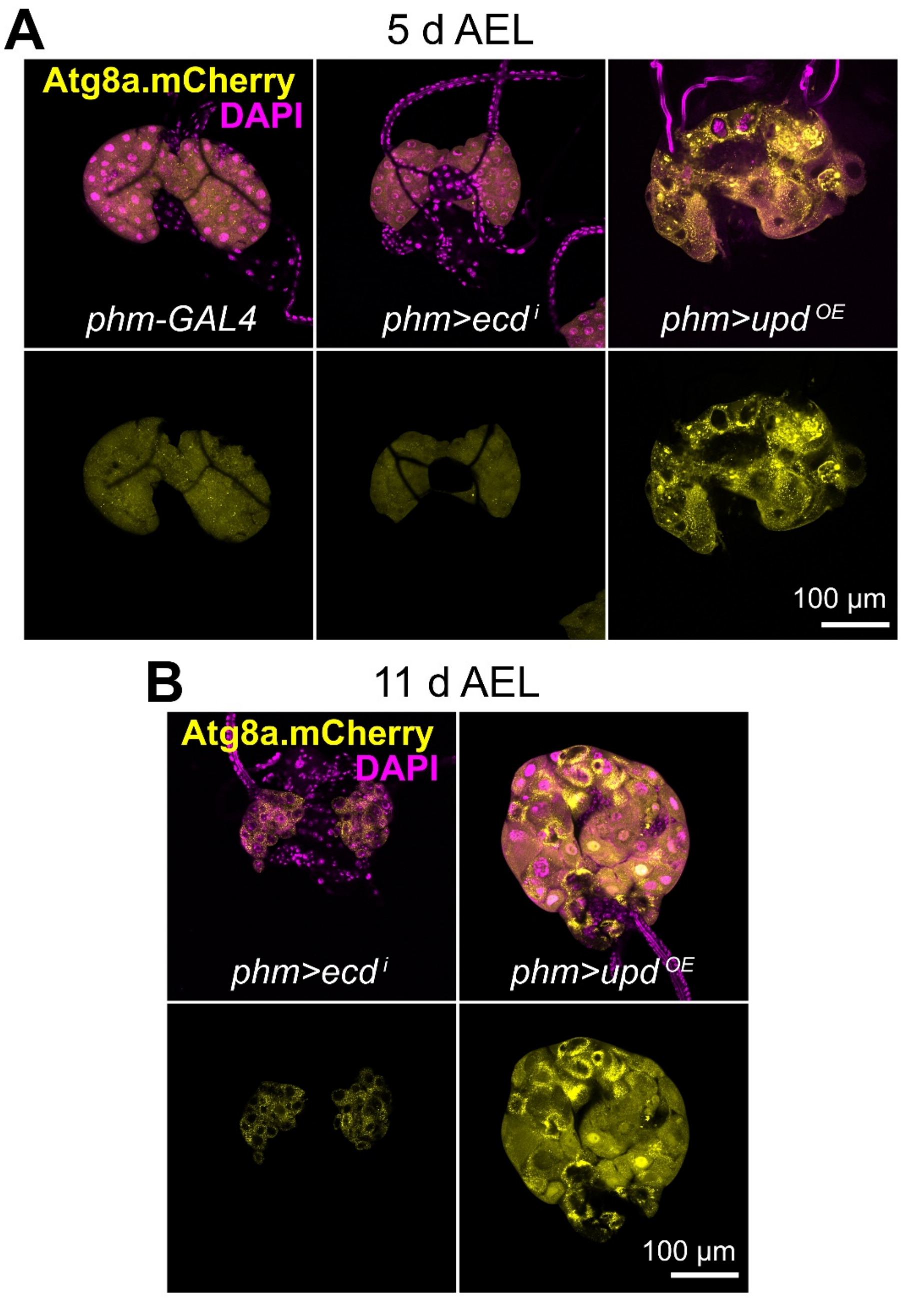

Fig. S4. PG hypertrophy and autophagy upon JAK/STAT hyperactivation precede developmental delay

(A) Autophagy marker Atg8.mCherry (yellow) in the PG of control *phm-GAL4* (left), *phm>ecd*^{i} (center) and *phm>upd*^{OE} (right) wL3 larvae, dissected 5 d AEL. Nuclei stained with DAPI (magenta). Hypertrophy and strong autophagy induction are seen in *phm>upd*^{OE} PG before pupation delay.

(B) Autophagy marker Atg8.mCherry (yellow) in the PG of *phm>ecd*^{i} (left) and *phm>upd*^{OE} (right) giant larvae. Nuclei stained with DAPI (magenta).

Disease Models & Mechanisms • Supplementary information

Table S1. Detailed genotypes. Genotypes of animals in all experiments, listed by figure.

Click here to download Table S1

Table S2. Original strains. Strains used in the study and their origin.

Click here to download Table S2
